# Supramolecular isomerism and structural flexibility in coordination networks sustained by cadmium rod building blocks†

**DOI:** 10.1039/d3ce00557g

**Published:** 2023-07-04

**Authors:** Yassin H. Andaloussi, Andrey A. Bezrukov, Debobroto Sensharma, Michael J. Zaworotko

**Affiliations:** a Department of Chemical Sciences, Bernal Institute, University of Limerick Limerick V94 T9PX Republic of Ireland xtal@ul.ie

## Abstract

Bifunctional N-donor carboxylate linkers generally afford **dia** and **sql** topology coordination networks of general formula ML_2_ that are based upon the MN_2_(CO_2_)_2_ molecular building block (MBB). Herein, we report on a new N-donor carboxylate linker, β-(3,4-pyridinedicarboximido)propionate (PyImPr), which afforded Cd(PyImPr)_2_*via* reaction of PyImPrH with Cd(acetate)_2_·2H_2_O. We observed that, depending upon whether Cd(PyImPr)_2_ was prepared by layering or solvothermal methods, 2D or 3D supramolecular isomers, respectively, of Cd(PyImPr)_2_ were isolated. Single crystal X-ray diffraction studies revealed that both supramolecular isomers are comprised of the same carboxylate bridged rod building block, RBB. We were interested to determine if the ethylene moiety of PyImPr could enable structural flexibility. Indeed, open-to-closed structural transformations occurred upon solvent removal for both phases, but they were found to be irreversible. A survey of the Cambridge Structural Database (CSD) was conducted to analyse the relative frequency of RBB topologies in related ML_2_ coordination networks in order to provide insight from a crystal engineering perspective.

## Abstract 2

Using the new bifunctional PyImPr linker and cadmium, two coordination networks, sustained by rod building blocks, form 2D or 3D supramolecular isomers that undergo open-to-closed phase transformations enabled by the flexible linker.

## Graphical abstract

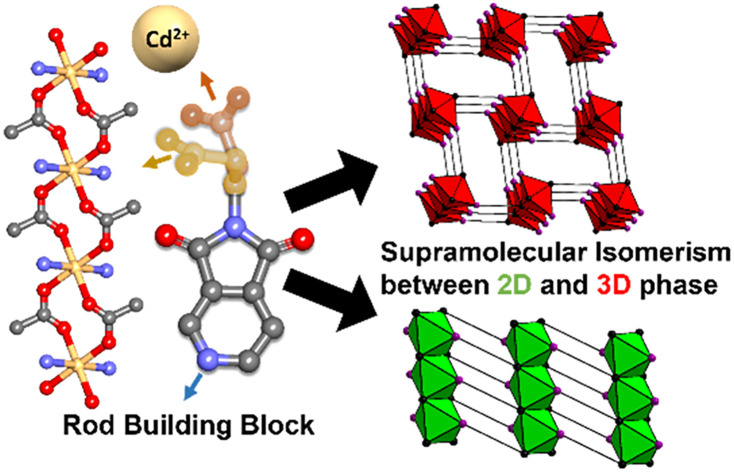

## Introduction

That crystal engineering has come of age is exemplified by the emergence of families of coordination networks (CNs) that are amenable to design by self-assembly involving metal cation or metal cluster molecular building blocks (MBBs)^1^ linked into 2D or 3D networks by organic and/or inorganic linkers.^2^ Interest in CNs has grown for several reasons, including the highly modular nature of the components, which offers chemical and structural diversity,^3^ and control over pore size and chemistry to enable systematic crystal engineering studies of structure/function relationships. CNs may be formed from MBBs based upon long established coordination environments such as “Werner complexes” of the general formula ML_4_X_2_.^4,5^ Such MBBs have been exploited to generate families of CNs with 2D **sql** (square lattice) topology sustained by the prototypal linker 4,4′-bipyridine.^6,7^

Metal–organic frameworks (MOFs) are a subset of CNs that feature potential voids.^2^ The rapid development of MOFs^8,9^ has resulted in properties of relevance to applications such as gas/vapour storage,^10,11^ gas/vapour separation,^12,13^ catalysis,^14^ proton conductivity^15^ and chiral resolution.^16^ The taxonomic classification of CNs^17^ can be a useful crystal engineering tool and relies in part on identifying the underlying connectivity, or topology of a CN.^18,19^ Topologies are typically denoted by 3-letter codes such as the frequently encountered **dia** (diamondoid)^20,21^ or **sql**^22^ nets and are archived in the Reticular Chemistry Structural Resource (RCSR) database.^23^

Ditopic linkers^24^ are typically comprised of (a) only N-donor groups (*e.g.* 4,4′-bipyridine); (b) only carboxylate groups (*e.g.* terephthalic acid); or (c) mixed functionality, especially N-donor and carboxylate groups (*e.g.* isonicotinic acid).^25^ Mixed N-donor carboxylate linkers are of interest to crystal engineering as they facilitate the generation of families of charge-neutral single-linker networks of ML_2_ stoichiometry (M = divalent metal ion, L = linker). This stoichiometry allows for targeting of CNs with **dia**^26–29^ or **sql**^30–33^ topologies, which, when porous, are well-studied platforms for the evaluation of properties relevant to gas storage and separation.^34–36^

**Scheme 1:**
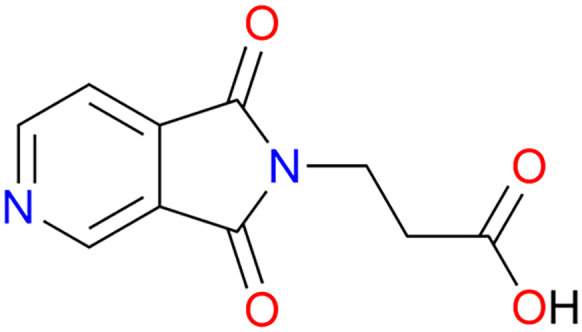
β-(3,4-Pyridinedicarboximido)propionic acid (PyImPrH).

Herein, we report a new member of the ML_2_ family based upon the previously unstudied bifunctional N-donor carboxylate linker β-(3,4-pyridinedicarboximido)propionic acid (PyImPrH, Scheme 1) and Cd(ii). PyImPrH, prepared by reaction of 3,4-pyridinedicarboxylic anhydride and β-alanine, possesses an ethylene spacer group which we anticipated would impart flexibility upon the resultant CNs. Two supramolecular isomers^37^ of Cd(PyImPr)_2_, Cd(PyImPr)_2_-**2D** and Cd(PyImPr)_2_-**hlz**, were isolated; their structural properties and phase transformations are reported herein along with crystal engineering insight into the topologies exhibited by ML_2_ structures, as addressed by Cambridge Structural Database (CSD) database mining.

## Experimental

### Materials and instrumentation

3,4-Pyridinedicarboxylic anhydride was purchased from Fluorochem while all other reagents and solvents were purchased from Sigma Aldrich. All reagents and solvents were used as received without further purification. Crystal structures were determined by single crystal X-ray diffraction (SCXRD) with Cu Kα radiation (*λ* = 1.5418 Å) on a Bruker D8 Quest fixed-chi diffractometer equipped with a Photon II detector and a nitrogen-flow Oxford Cryosystem attachment. Data was indexed, integrated, and scaled in APEX4.^38^ Absorption corrections were performed by the multi-scan method using SADABS.^39^ Space groups were determined using XPREP,^40^ as implemented in APEX4. The SHELX-2014 program package, implemented in OLEX2 (ref. 41) v1.5 was used for structure solution and refinement. Structures were solved using the intrinsic phasing method (SHELXT)^42^ and refined with SHELXL^43^ using the least-squares method. Non-hydrogen atoms were refined anisotropically. Hydrogen atoms were positioned from the molecular geometry at idealised locations and assigned isotropic thermal parameters depending on the equivalent displacement parameters of their carriers. The crystal structure of Cd(PyImPr)_2_-2D-α was further refined using the OLEX2 implementation of BYPASS (a.k.a. SQUEEZE^44^) to remove the contribution of disordered solvent molecules to the structure factor. Crystallographic data have been deposited into the Cambridge Crystallographic Data Centre (CCDC 2241486–2241489).

Powder X-ray diffraction (PXRD) experiments were conducted using microcrystalline samples on a PANalytical Empyrean diffractometer (40 kV; 40 mA; CuKα_1,2_*λ* = 1.5418 Å) in Bragg–Brentano geometry. Powder patterns were calculated from SCXRD structures using Mercury.^45^ Thermogravimetric analyses were performed under N_2_ flow using a TA Instruments Q50 system. A sample was loaded into an aluminium sample pan and heated at 10 °C min^−1^ from room temperature to 400 °C. Differential scanning calorimetry was carried out using a TA Instruments Q2000 differential scanning calorimeter. Samples were prepared by crimping the sample pan and lid (a pin hole was placed in the lid to prevent pressure build-up). A reference pan was prepared in the same manner for each analysis. The sample and reference pans were heated at 10 °C min^−1^ from room temperature to 400 °C and so the heat flow, relative to the reference, was measured as a function of time and temperature under a controlled atmosphere. N_2_ gas flowing at a rate of 50 mL min^−1^ was used to purge the furnace (Fig. S21–S24†).

### β-(3,4-Pyridinedicarboximido)propionic acid (PyImPrH)

β-(3,4-Pyridinedicarboximido)propionic acid (PyImPrH) was synthesised following a procedure adapted from Perillo *et al.*^46^ 3,4-pyridinedicarboxylic anhydride (2.00 g, 13.41 mmol, 1 eq.) and β-alanine (1.32 g, 14.76 mmol, 1.1 eq.) was stirred in 13.4 mL of DMF for 2.5 h at 110 °C. After being allowed to cool to RT, 60 mL of distilled water was added and the solution was allowed to stir for 1 h as the product formed as colourless crystals, which were then vacuum filtered (2.09 g, 71% yield).

### Cd(PyImPr)_2_-**2D**

Cd(PyImPr)_2_-**2D**-α was formed by a layering procedure in which 4 mL of *p*-xylene buffer was carefully placed over 4 mL of a 17 : 3 DCM : DMF (v : v) solution of PyImPrH (44.0 mg, 0.200 mmol, 1 eq.) in a test tube. Above these layers, cadmium acetate dihydrate (53.3 mg, 0.200 mmol, 1 eq.) dissolved in 4 mL MeOH was carefully placed. After 11 days, colourless crystals formed at the MeOH/*p*-xylene boundary and were harvested from the test tube wall, vacuum filtered, and washed with a small quantity of MeOH (40.4 mg, 69% yield based on (Cd(PyImPr)_2_·MeOH)), (average yield over 10 identical experiments). When heated to 60 °C for 24 h, or when exposed to vacuum for 24 h, or when left at RT for several weeks, Cd(PyImPr)_2_-**2D**-α converted to a closed phase, Cd(PyImPr)_2_-**2D**-β, through a single-crystal-to-single-crystal transformation.

### Cd(PyImPr)_2_-**hlz**

Cd(PyImPr)_2_-**hlz**-α was formed by dissolving PyImPrH (110 mg, 0.500 mmol, 1 eq.) in 5 mL DMF, adding cadmium acetate dihydrate (66.6 mg, 0.250 mmol, 0.5 eq.) and heating at 60 °C for 48 h. The large colourless crystals then formed were harvested by vacuum filtration and washed with small quantities of DMF (136.8 mg, 79% yield based on (Cd(PyImPr)_2_·2DMF)). When heated to 105 °C for 24 h Cd(PyImPr)_2_-**hlz**-α converted to a closed phase, Cd(PyImPr)_2_-**hlz**-β, in a single-crystal-to-single-crystal transformation.

## Results

### Cd(PyImPr)_2_-**2D**

**Fig. 1:**
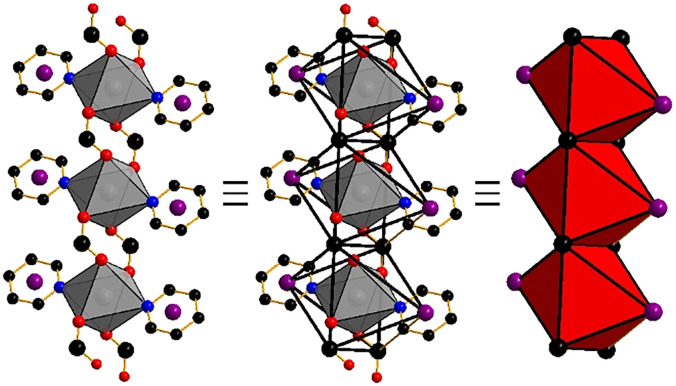
The cadmium carboxylate RBB in Cd(PyImPr)_2_. 6-Coordinate cadmium cations (gray) are bound to μ_2_-(O,O′) carboxylate and pyridine groups. The RBB may be formed from points of extension on the carboxylate carbon (black) and the pyridine centroid (purple). Points of extension link to form opposite edge-sharing octahedra (right).

**Fig. 2:**
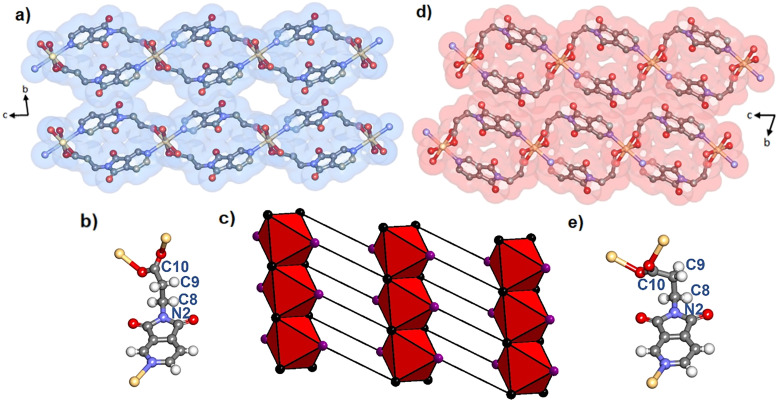
Crystal structures of Cd(PyImPr)_2_-**2D**-α (a) and Cd(PyImPr)_2_-**2D**-β (d) as viewed along the *a*-axis along with a VdW surface. Hydrogen atoms have been omitted for clarity. (b) and (e) show the antiperiplanar and *gauche* conformation of the PyImPr linker in Cd(PyImPr)_2_-**2D**-α and Cd(PyImPr)_2_-**2D**-β, respectively, while (c) shows the net of Cd(PyImPr)_2_-**2D**-α formed from edge-sharing octahedra with carboxylate carbons shown in black and pyridine centroids show in purple.

A crystal of Cd(PyImPr)_2_-**2D**-α studied by single crystal X-ray diffraction (SCXRD) revealed that it had adopted the space group *P*1̄ and the expected ML_2_ composition (Fig. S1 and S2†). Bulk phase purity was confirmed by powder X-ray diffraction (PXRD, Fig. S15†). As detailed in Fig. 1, the crystal structure is formed from rod building blocks (RBBs) that are sustained by μ_2_-(O,O′) carboxylate groups. These RBBs lie parallel to the *a*-axis and are linked in a spiro fashion by two linkers to adjacent RBBs (Fig. 2a). The resulting sheets lie along the *ac* plane and exhibit a previously unassigned (5,8)-c topology (referred to hereinafter as **2D-1**) with point symbols (3^4^·4^4^·5^2^)(3^8^·4^10^·5^7^·6^3^), respectively (Fig. 2c). The stacking mode of the undulating sheets creates an interlayer extrinsic void space of 14.5% in which disordered MeOH molecules reside, as indicated by the residual electron density. The aliphatic region of the linker allows for two likely conformations: antiperiplanar, with the bulkier aromatic and carboxylate groups pointing away from each other; and *gauche*, with the bulky groups in closer contact. The N2–C8–C9–C10 torsion angle of 158.2(3)° is consistent with the less sterically hindered antiperiplanar conformation (Fig. 2b). As a result, the linkers within each layer exhibit short-contact 2.819(4) Å repulsive interactions between the intra-network imide O1 oxygen groups (Fig. S3†). This contrasts with the inter-network interactions between imide carbonyl O2 oxygen and C7 carbon atoms (3.174(6) Å), and C–H⋯O hydrogen bonds between the O2 carbonyl oxygen and the C5 pyridyl carbon (O2⋯C5 = 3.477(5) Å).

Desolvation by heating, exposure to vacuum, or drying in air was found by PXRD (Fig. S15†), TGA (Fig. S19†), and SCXRD to induce a phase transformation of the as-synthesised phase Cd(PyImPr)_2_-**2D**-α, to the non-porous phase Cd(PyImPr)_2_-**2D**-β (Fig. 2d and S4 and S5†). TGA data revealed a weight loss onset at *ca.* 50 °C of 11.0% consistent with a loss of two MeOH molecules per formula unit (calculated 11.6%). DSC displayed a matching endothermic peak and an additional endothermic peak from thermal decomposition at 283 °C (Fig. S21 and S22†). This transformation was accompanied by a conformational change of the PyImPr linker from antiperiplanar to *gauche*, with the torsion angle changing from 158.2(3)° to 80.3(9)° (Fig. 2e). The conformational change resulted in the spiro-shaped rings within the nets becoming more rounded. The layers shifted with respect to one another such that the extrinsic void space between RBBs was reduced, with the unit cell volume decreasing by 11.5%. We note that this conformation change resulted in an increased distance between O1 carbonyl oxygens to 3.710(9) Å, thus diminishing the repulsive interactions seen in the open phase (Fig. S6†). Between the 2D layers, the effect of the conformational change was to remove the close interactions between Cd(PyImPr)_2_-**2D**-α carbonyl groups, eliciting C–H⋯O hydrogen bonding interactions (3.235(11) Å between O2 and C5, and 3.581(10) Å between O2 and C9).

### Cd(PyImPr)_2_-**hlz**

**Fig. 3:**
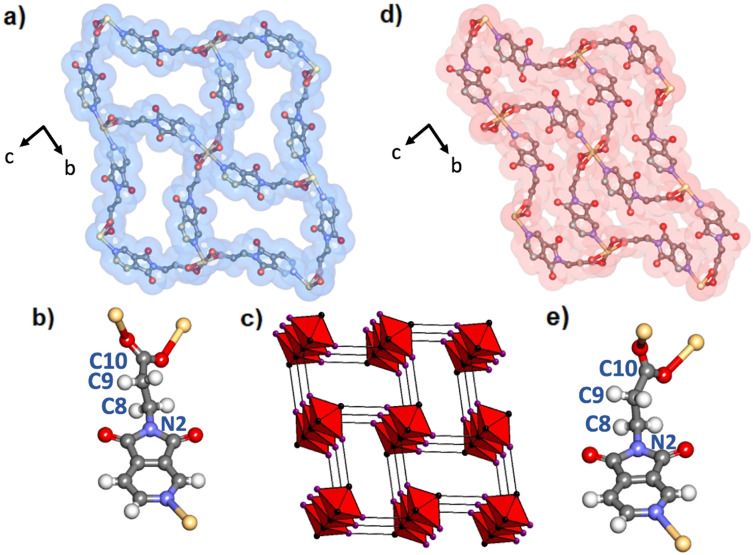
Crystal structures of Cd(PyImPr)_2_-**hlz**-α (a) and Cd(PyImPr)_2_-**hlz**-β (d) as viewed along the *a*-axis along with a VdW surface. Pore DMF molecules in (a) and hydrogen atoms in (a) and (d) have been omitted for clarity. (b) and (e) show the conformation and coordination of the PyImPr linker in Cd(PyImPr)_2_-**hlz**-α and Cd(PyImPr)_2_-**hlz**-β, respectively, while (c) shows the net of Cd(PyImPr)_2_-**hlz**-α at an offset from the *a*-axis formed from edge-sharing octahedra with carboxylate carbons shown in black and pyridine centroids show in purple.

Heating of PyImPrH with Cd(acetate)_2_·2H_2_O in DMF afforded wedge-shaped monoclinic crystals. SCXRD revealed a different form of Cd(PyImPr)_2_ in space group *P*2_1_/*c* (Fig. S7–S9†). Bulk phase purity was confirmed by powder X-ray diffraction (PXRD) (Fig. S16†). The same RBBs as in Cd(PyImPr)_2_-**2D** had formed (Fig. 1), but each alternating linker along the RBB chain connects separate RBBs, enabling the structure to propagate in three dimensions (Fig. 3a). The result is a supramolecular isomer with (5,8)-c **hlz** topology and point symbols (3^4^·4^2^·5^4^)(3^8^·4^8^·5^6^·6^5^·7) (Fig. 3c). Cd(PyImPr)_2_-**hlz**-α exhibits rectangular-shaped 1D pores containing two ordered molecules of DMF in a void space of 33%. The PyImPr linker adopted an antiperiplanar conformation with a torsion angle of 176.7(2)° about the N2–C8–C9–C10 bonds (Fig. 3b). Each RBB is rotated 24° about the adjacent RBB chains as measured by planes formed from the pyridine centroids along each rod (Fig. S8†). Multiple short-contact interactions were observed between the framework and the DMF molecules in the pore, including: 1) a 3.093 Å π⋯O between the DMF oxygen atom and the pyridine ring centroid; 2) two C–H⋯O hydrogen bonds (3.381(4) Å between O1 and C11 and 3.567(4) Å between O5 and C9) (Fig. S10†).

As indicated by TGA and DSC, Cd(PyImPr)_2_-**hlz**-α desolvated from *ca.* 85 °C (Fig. S20, S23 and S24†) with a weight loss of 21.3% and a corresponding endothermic peak consistent with the loss of two DMF molecules (calculated 21.0%) as well as a subsequent endothermic peak from thermal decomposition at 274 °C. When single crystals were heated at 105 °C for 24 h, the clear crystals were observed to turn opaque but remained crystalline (Fig. S11–S13†). SCXRD analysis revealed a non-porous phase, Cd(PyImPr)_2_-**hlz**-β, with 30% smaller unit cell volume and the *c*-axis reduced from 15.0256(3) Å to 10.9159(5) Å (Fig. 3d). The space group and **hlz** topology were retained, however the aliphatic N2–C8–C9–C10 torsion angle was reduced from 176.7(2)° to 151.2(8)° (Fig. 3e). This modest change in angle had the effect of increasing the curvature of each edge of the rectangular pore walls, resulting in a short-contact of 3.202(11) Å between the imide carbonyl O2 and the carboxylate C10 (Fig. S14†). This also resulted in a reduction in the angle made between adjacent RBBs to 6.7° as measured by the proximal planes formed from the pyridine centroids along each rod (Fig. S12†). Bulk phase purity was confirmed by powder X-ray diffraction (PXRD) (Fig. S16†).

## Discussion

It has previously been reported that as-synthesised porous CNs that contain solvent (α phases) can undergo single-crystal-to-single-crystal transformation to desolvated non-porous phases (β phases) and that such transformations can be reversible.^47,48^ Such “switching” CNs could be relevant to gas or vapour storage and separation. Unfortunately, neither of the β phases of Cd(PyImPr)_2_ reported herein were found to revert to the open phases once formed, despite exposure to a range of guests such as DMF, MeOH or the respective mother liquors (Fig. S17 and S18†). In the case of Cd(PyImPr)_2_-**2D**, this may be a consequence of the *gauche*-linker being better able to accommodate the oval-shaped structure with reduced repulsions between the intra-network carbonyl groups. In the case of Cd(PyImPr)_2_-**hlz**, the lack of reversibility was less clear, however the close contact of imide carbonyl and carboxylate groups may act as a barrier to solvation.

The ability of certain RBBs to exhibit structural flexibility is well known, with breathing behaviour reported, *e.g.* in MIL-47(V),^49^ and switching between open and closed phases, *e.g.* in MIL-53(Sc).^50^ Therefore, the scope for applying RBB-based MOFs remains promising. Furthermore, RBB structures have often been thought desirable due to their repeat relatively short repeat distances hindering the possibility of interpenetration, *i.e.* “forbidden catenation”.^51^ This enables design and synthesis of variants with progressively larger surface areas, as in MOF-74 and its derivatives,^52,53^ without the risk of network interpenetration and reduced surface areas.

Edge-sharing octahedral RBB nets as observed in Cd(PyImPr)_2_ have been reported in other ML_2_ structures involving N-donor carboxylate linkers and bivalent metal ions.^54^ However, these structures are almost exclusively identified in the literature with topologies wherein the N-donor carboxylate linkers are considered to function as 3-connected nodes and the metal sites as 6-connected nodes. This approach, therefore, fails to account for the existence of the RBB (Fig. S29†). This is especially the case when automatic topology determination software such as TopCryst^55^ or the TOPOS TTO database^56^ is used. Furthermore, the systematic analysis of reported RBBs is hindered by shortcomings in the search function of the ConQuest^57^ CSD software which causes some periodic structures to not be readily discoverable (Fig. S25†).

**Fig. 4:**
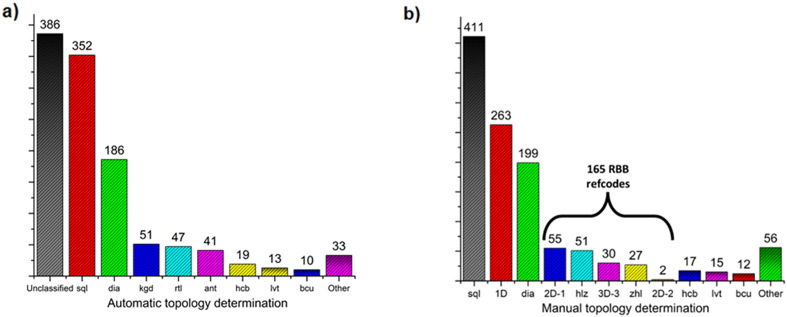
Number of N-donor carboxylate refcodes belonging to each topology: a) after automatic topology determination; b) after manual topology determination.

In order to enable a systematic analysis of RBBs among previously reported ML_2_ frameworks, CSD database mining was performed, followed by topology determination (Fig. 4, see ESI† for details Fig. S26–S28). Among the 1138 identified ML_2_ structures based on divalent metal and bifunctional N-donor carboxylate linkers (Fig. 4a), 352 and 186 were automatically identified (by the TTO database) to exhibit 4-connected **sql** and **dia** topologies, respectively. Less common topologies found were the 51, 47, and 41 refcodes assigned to the binodal (3,6)-connected **kgd**, **rtl**, and **ant** topologies, respectively. However, using the method introduced by O'Keeffe, Yaghi *et al.*^58,59^ enables classification of RBB network topology by identifying appropriate points of extension. This, however, is a process not readily performed automatically by topology determination software. As such, the topology of these 1138 ML_2_ structures were determined by manual inspection, allowing for the appropriate MBB/RBB nodes to be determined, followed by net simplification and topology determination through ToposPro^56^ with the results shown in Fig. 4b.

From these results, it can be determined that **sql** structures are more common for this class of linker, followed by 1D structures, then **dia**, **2D-1**, **hlz**, “**3D-3**”,^60^**zhl**, **hcb**, **lvt**, **bcu**, **2D-2** and other less common topologies. As seen in Fig. S30 and S31,† all refcodes automatically determined to be **rtl** or **kgd** were found to be **hlz** or **2D-1**, respectively, while structures automatically determined as **ant** were manually assigned as either **zhl** or **3D-3**. In the case of **zhl** or **3D-3** topology determinations, this difference in topology is evident from the differing coordination environments; **3D-3** structures typically exhibit N-donor groups coordinating on opposite sides of a metal centre, resulting in an RBB composed of opposite-edge sharing octahedra; in **zhl** structures the N-donor groups are adjacent, resulting in RBBs composed of adjacent edge-sharing octahedra (Fig. S32 and S33†).

**Scheme 2:**
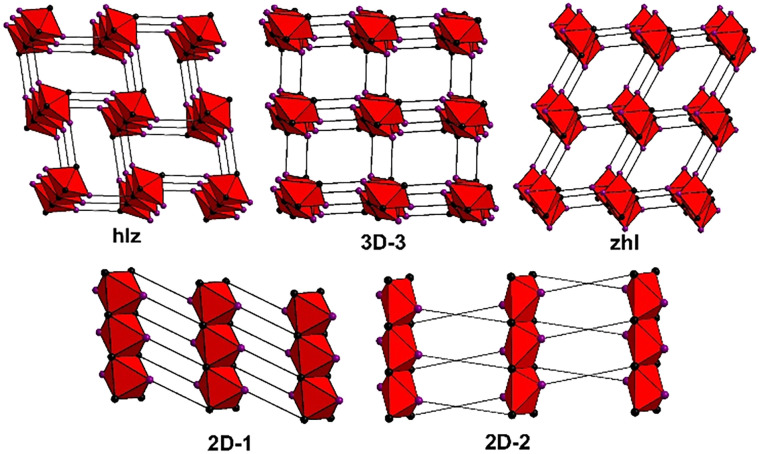
Possible RBB topologies of ML_2_ N-donor carboxylates.

Such distinctions concerning RBB connectivity is lost when the structure is described in terms of the **ant** topology. In summary, 3D RBB topologies with (5,8)-connectivity such as **hlz**, **zhl** and the previously unassigned “**3D-3**”,^60^ (Scheme 2) are typically identified as the (3,6)-c **rtl** or **ant** topologies while the (5,8)-c **2D-1** topology is typically identified as a **kgd** topology. Altogether, 165 refcodes with RBB topologies were identified and a full list is available in Table S2.†

From our systematic review of RBBs in ML_2_ frameworks, we found the supramolecular isomerism exhibited in Cd(PyImPr)_2_ to be rare. Within the ML_2_ structures found using our database mining approach, only a handful of examples exist of linkers that form multiple RBB topologies: 1*H*-tetrazol-1-ylacetic acid which forms **hlz**^61,62^ and the **3D-3** (ref. 63 and 64) topologies or 5-(3-pyridyl)-1,3,4-oxadiazole-2-thioacetic acid that forms the **hlz**^65^ or **zhl**^66^ topologies. Notably, all these examples showed isomerism only between different 3D networks, while Cd(PyImPr)_2_ was found to exhibit both 3D and 2D frameworks.

## Conclusions

RBBs enable unique coordination network topologies that expand on the most commonly observed CN structural types. Through the analysis of 1138 ML_2_ refcodes archived in the CSD reported herein, we have established that RBBs are present in 165 entries. In the case herein, the N-donor carboxylate linker PyImPr was found to afford 1D RBBs that cross-link to form either 2D sheet or 3D framework structures, depending on the synthetic conditions. The nature of the flexible linker enabled both of these structures to undergo irreversible structural transformations when desolvated, *i.e.* from an open phase to a closed phase. That Cd(PyImPr)_2_ formed supramolecular isomers and transformed irreversibly to closed phases are perhaps unexpected. This work highlights that, despite ML_2_ structures offering such a simple and predictable composition, the crystal engineering principles governing the adoption of RBB structures, and their phase transformations, remain largely understudied and are therefore of interest for further study given the potential utility of such structures.

## Conflicts of interest

There are no conflicts to declare.

## Supplementary Material

CE-025-D3CE00557G-s001

CE-025-D3CE00557G-s002
