# Supplementary material for: Supramolecular isomerism and structural flexibility in coordination networks sustained by cadmium rod building blocks
Source: CrystEngComm. 2023 Jul 4;25(29):4175–81. doi: 10.1039/d3ce00557g (PMC10364239; doi:10.1039/d3ce00557g)
Supplement: CE-025-D3CE00557G-s001 [file CE-025-D3CE00557G-s001.pdf]

Electronic Supplementary Material (ESI) for CrystEngComm.
This journal is © The Royal Society of Chemistry 2023

Supporting information

Supramolecular Isomerism and Structural Flexibility in Coordination Networks Sustained by Cadmium Rod Building Blocks

Yassin H. Andaloussi,[a] *Andrey A. Bezrukov,*[a] *Debobroto Sensharma,*[a] *and Michael J. Zaworotko*[a] *

[a] Department of Chemical Sciences and Bernal Institute, University of Limerick, Co. Limerick, Y94T9PX, Ireland.

*Email: Michael.Zaworotko@ul.ie

Contents

Crystal structures 2
Cd(PyImPr)$_2$-**2D**-α 2
Cd(PyImPr)$_2$-**2D**-β 3
Cd(PyImPr)$_2$-**hlz**-α 5
Cd(PyImPr)$_2$-**hlz**-β 7
PXRD Data 9
Cd(PyImPr)$_2$-**2D** 9
Cd(PyImPr)$_2$-**hlz** 10
Cd(PyImPr)$_2$-**2D** soaking experiments 11
Cd(PyImPr)$_2$-**hlz** soaking experiments 12
TGA Data 13
Cd(PyImPr)$_2$-**2D** 13
Cd(PyImPr)$_2$-**hlz** 13
DSC Data 14
Cd(PyImPr)$_2$-**2D**-α 14
Cd(PyImPr)$_2$-**2D**-β 14
Cd(PyImPr)$_2$-**hlz**-α 15
Cd(PyImPr)$_2$-**hlz**-β 15
Crystallographic table 16
Database mining 17
Mining Methodology 18
Datamining results 23
ML_2 structures based on N-donor carboxylate linker 23
3D N-donor carboxylate RBB topologies 24
2D N-donor carboxylate RBB topologies 25
ML_2 structures based on N-donor carboxylate linkers with edge-sharing octahedra RBBs 26
References 31

Crystal structures

Cd(PyImPr)$_2$-**2D**-α

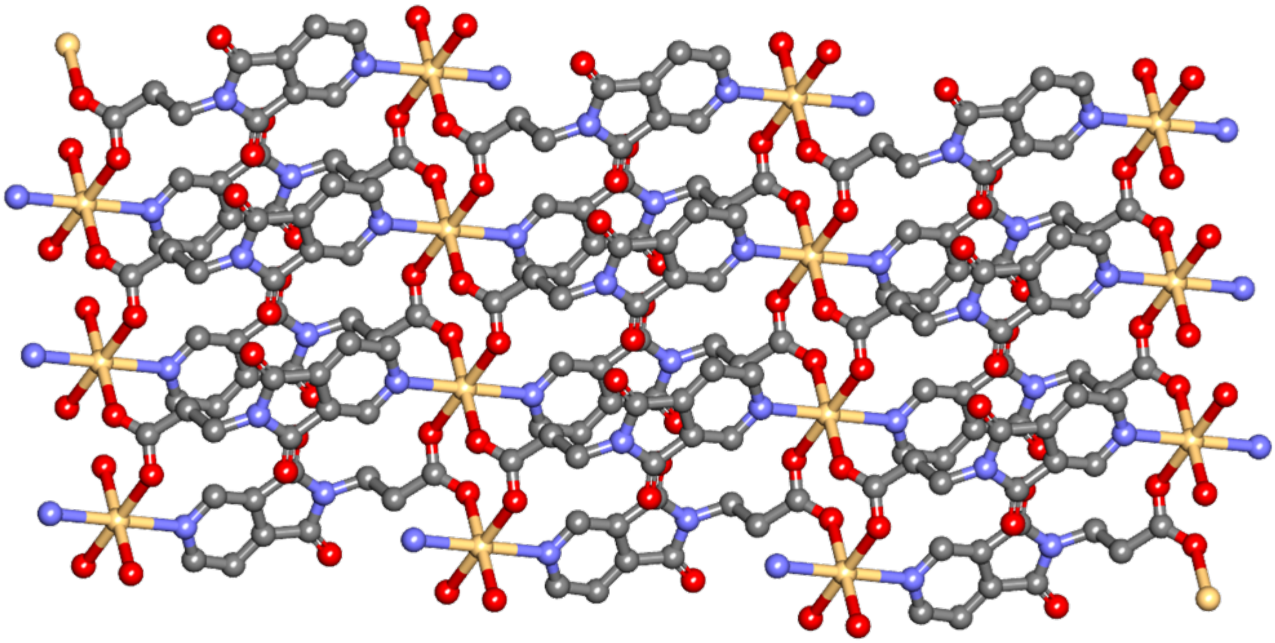

Figure S1: Cd(PyImPr)$_2$-**2D**-α as viewed along the c-axis. Hydrogen atoms have been omitted for clarity.

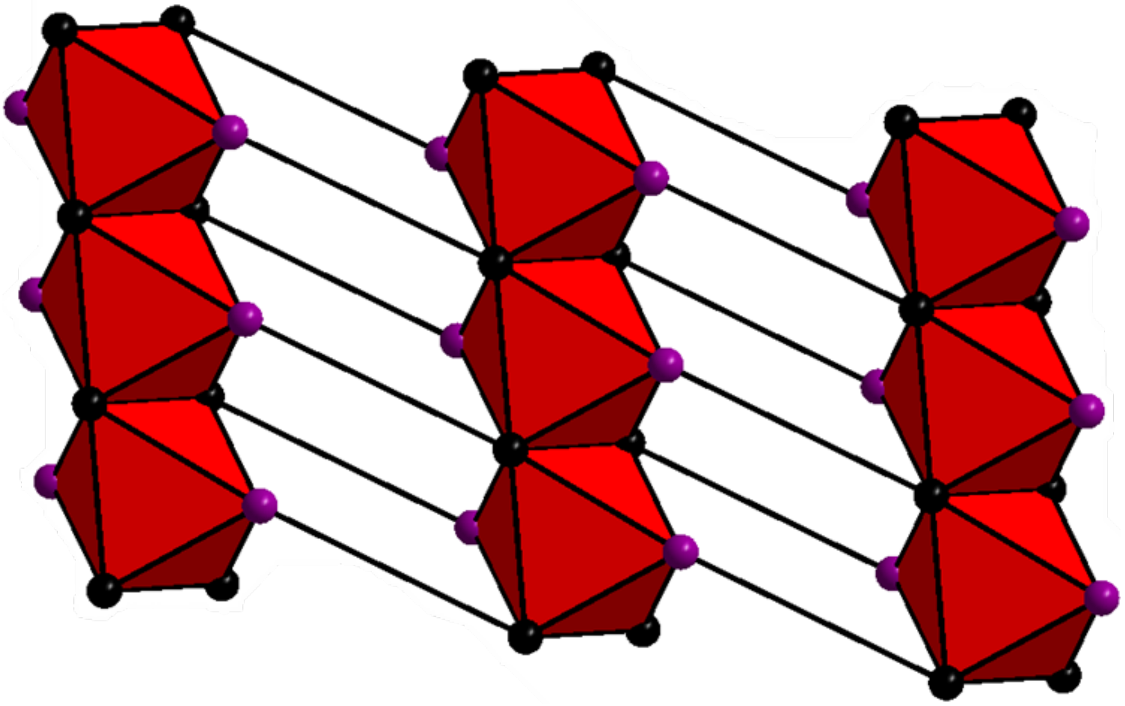

Figure S2: Topological representation of Cd(PyImPr)$_2$-**2D**-α with the points of extension – the C10 carboxylate carbon and the pyridine centroid in black and purple, respectively. These link to form edge-sharing octahedra shown in red that form 2D sheets.

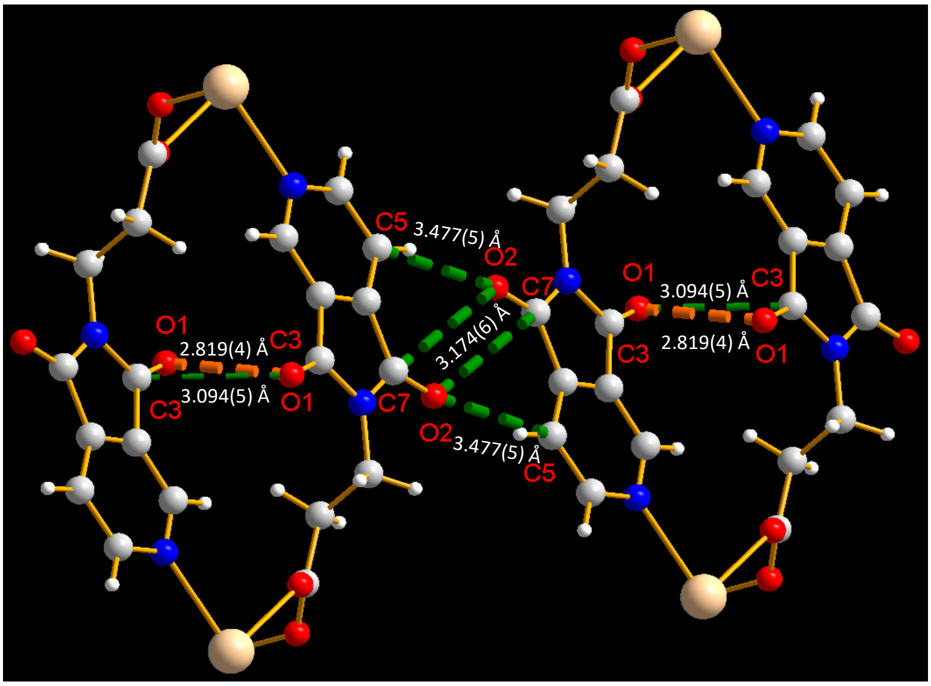

Figure S3: Select close interactions within and between the 2D nets in Cd(PyImPr)$_2$-**2D**-α.

Cd(PyImPr)$_2$-**2D**-β

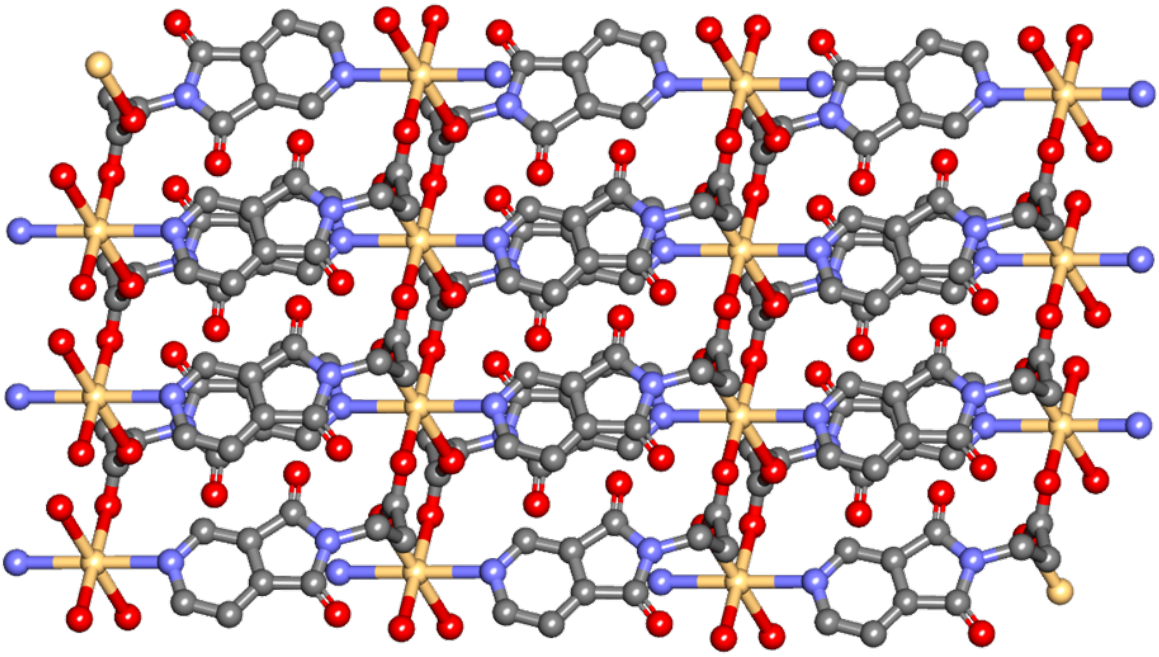

Figure S4: Cd(PyImPr)$_2$-**2D**-β as viewed along the c-axis. Hydrogen atoms have been omitted for clarity.

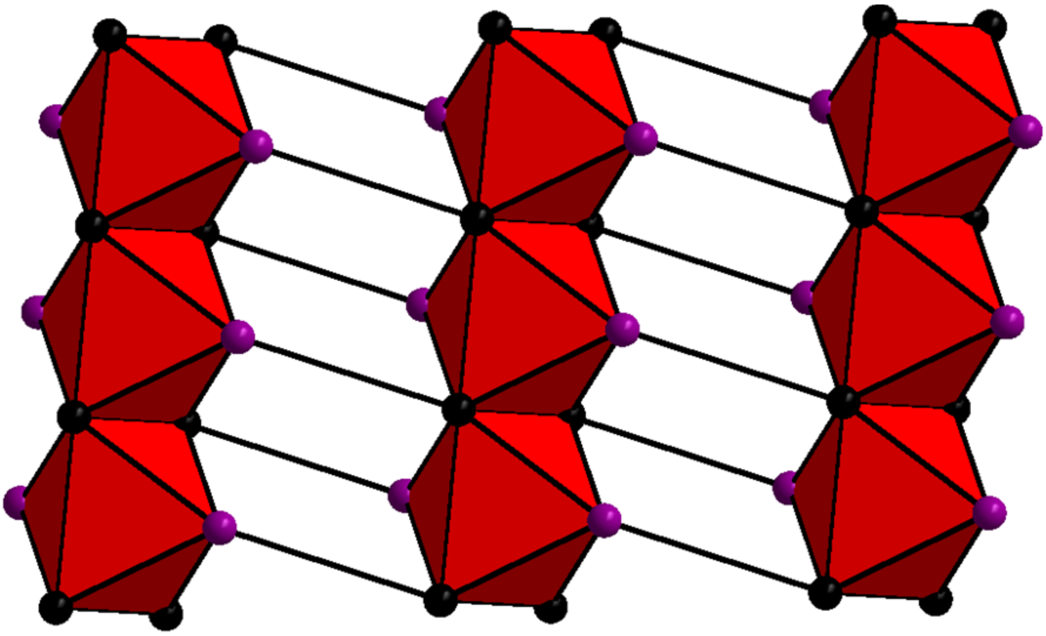

Figure S5: Topological representation of Cd(PyImPr)$_2$-**2D**-β with the points of extension – the C10 carboxylate carbon and the pyridine centroid in black and purple, respectively. These link to form edge-sharing octahedra shown in red that form 2D sheets.

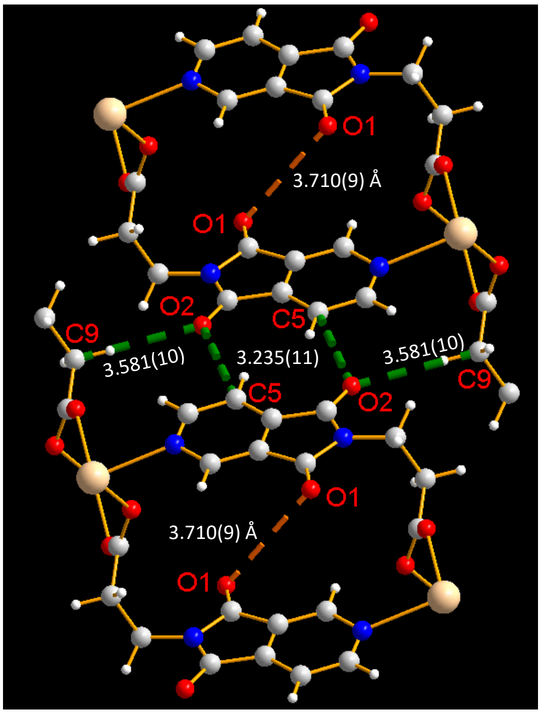

Figure S6: Select close interactions within and between the 2D nets in Cd(PyImPr)$_2$-**2D**-β.

Cd(PyImPr)$_2$-**hlz**-α

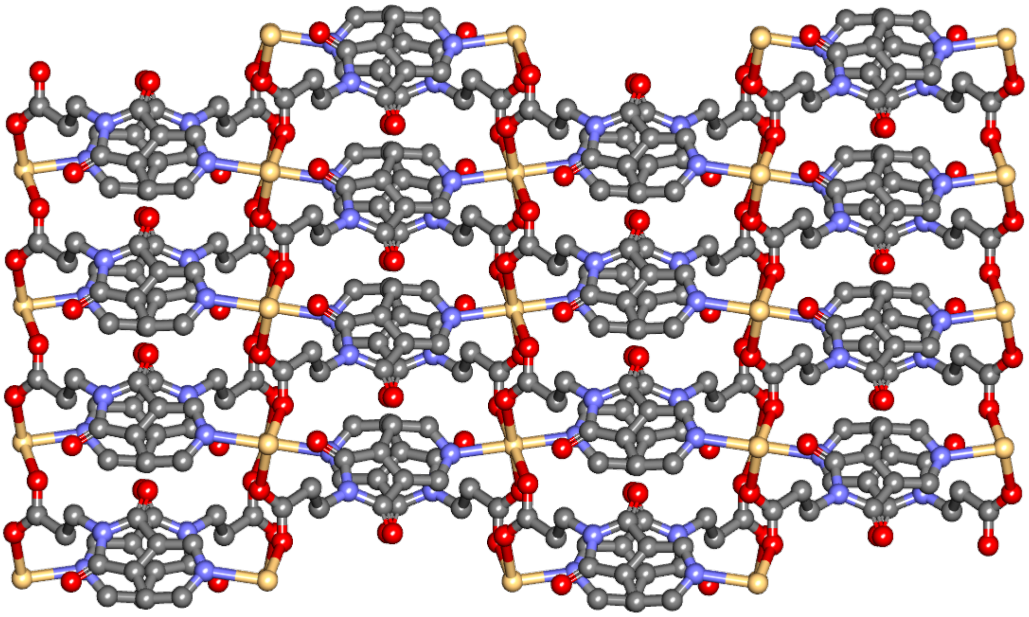

Figure S7: Cd(PyImPr)$_2$-**hlz**-α as viewed along the c-axis. Hydrogen atoms have been omitted for clarity.

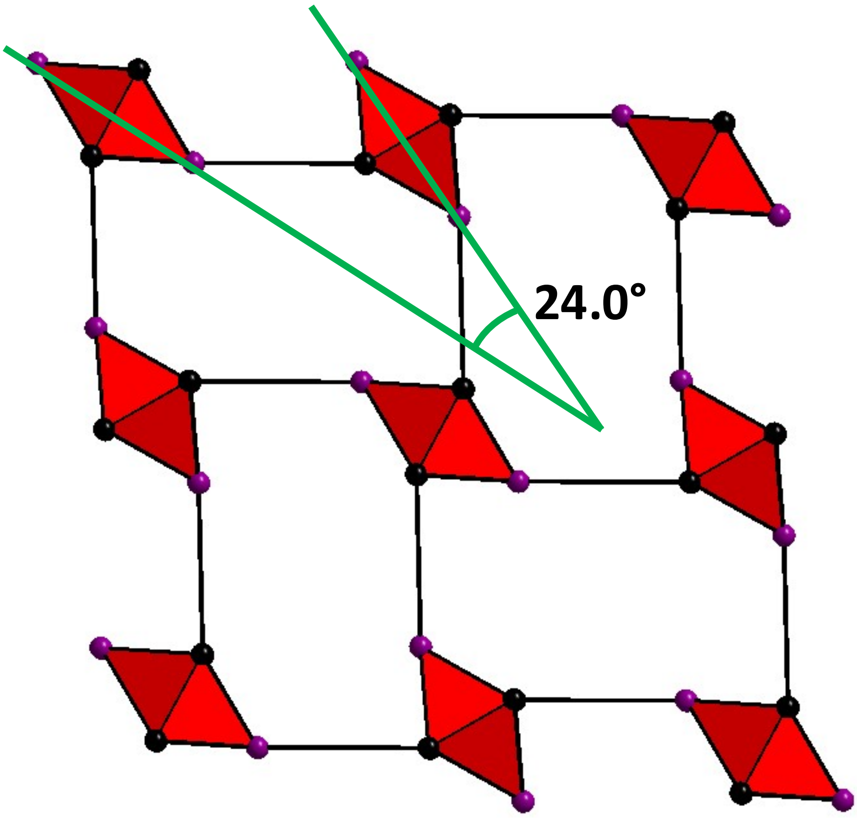

Figure S8: Topological representation of Cd(PyImPr)$_2$-**hlz**-α along the a-axis with the points of extension – the C10 carboxylate carbon and the pyridine centroid in black and purple, respectively. These link to form edge-sharing octahedra shown in red that form a 3D net resulting in the **hlz** topology. The angle formed between RBB units as measured using planes formed from the pyridine centroid points of extension is also shown.

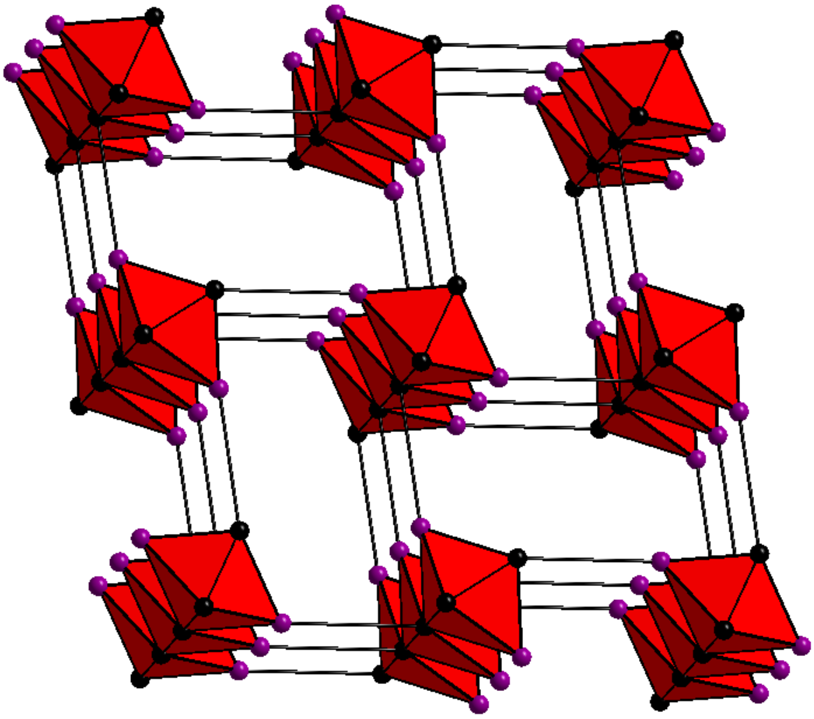

Figure S9: Topological representation of Cd(PyImPr)$_2$-**hlz**-α at an offset from the a-axis with the points of extension – the C10 carboxylate carbon and the pyridine centroid in black and purple, respectively. These link to form edge-sharing octahedra shown in red that form a 3D net resulting in the **hlz** topology.

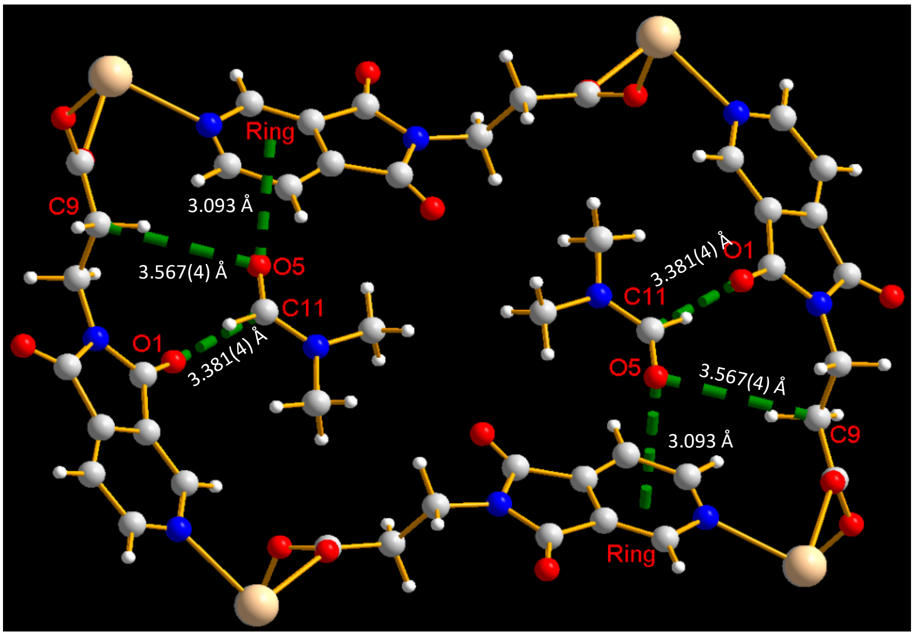

Figure S10: Select close interactions between pore DMF molecules and the 3D framework in Cd(PyImPr)$_2$-**hlz**-α.

Cd(PyImPr)$_2$-**hlz**-β

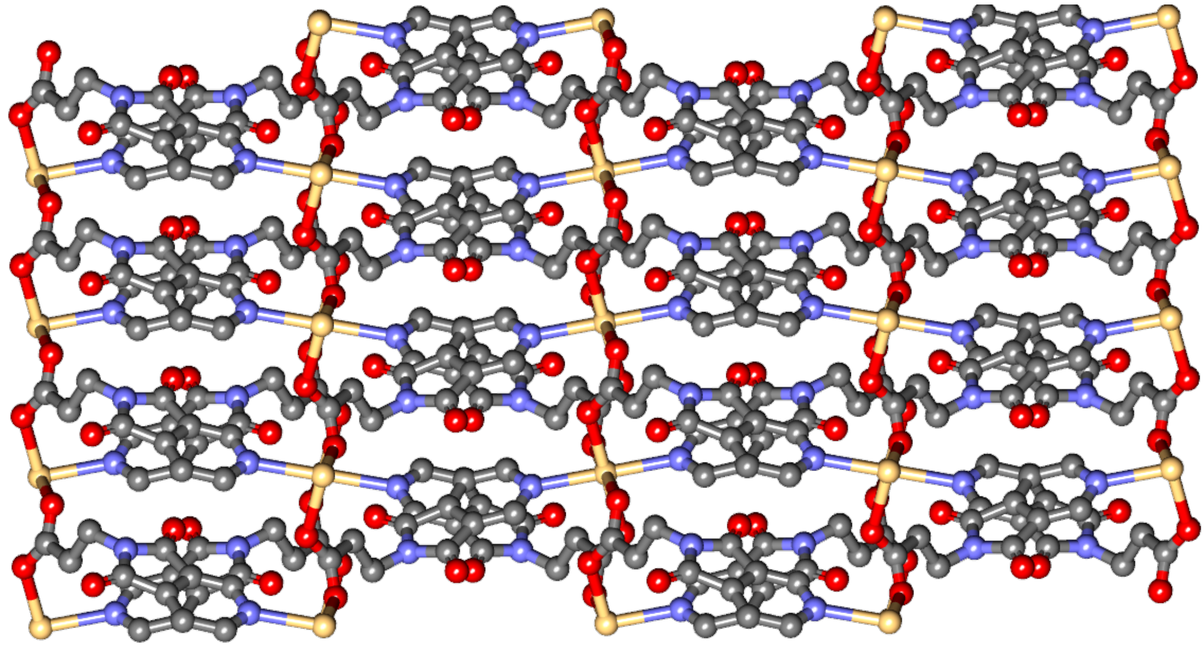

Figure S11: Cd(PyImPr)$_2$-**hlz**-β as viewed along the c-axis. Hydrogen atoms have been omitted for clarity.

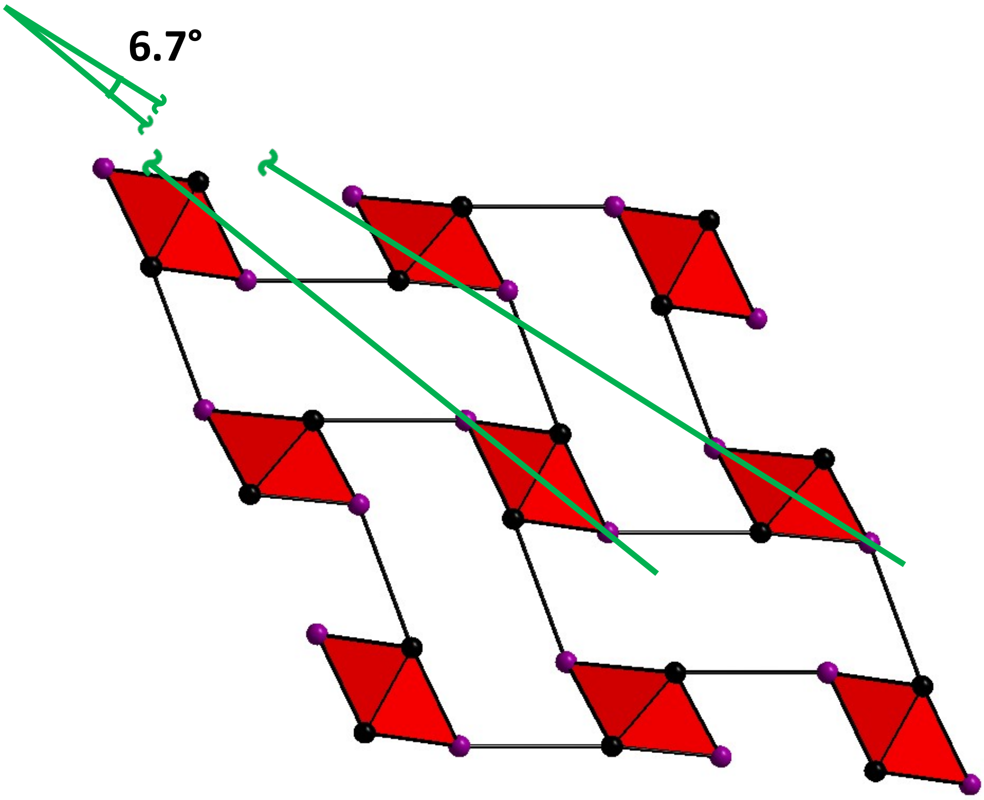

Figure S12: Topological representation of Cd(PyImPr)$_2$-**hlz**-β along the a-axis with the points of extension – the C10 carboxylate carbon and the pyridine centroid in black and purple, respectively. These link to form edge-sharing octahedra shown in red that form a 3D net resulting in the **hlz** topology. The angle formed between RBB units as measured using planes formed from the pyridine centroid points of extension is also shown.

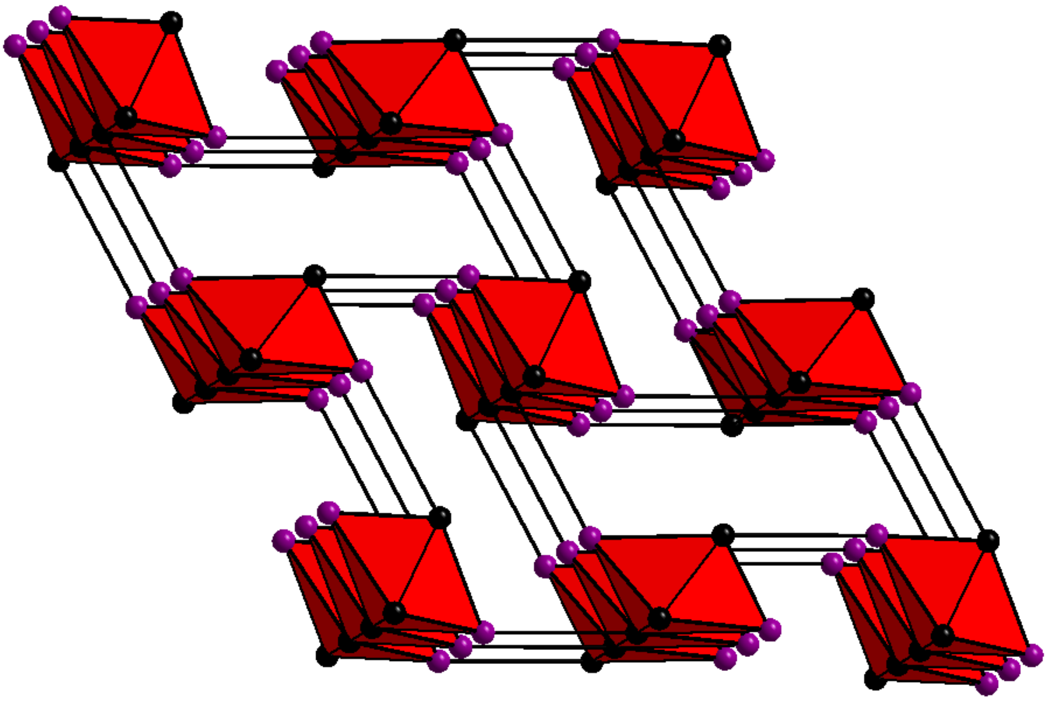

Figure S13: Topological representation of Cd(PyImPr)$_2$-**hlz**-β at an offset from the a-axis with the points of extension – the C10 carboxylate carbon and the pyridine centroid in black and purple, respectively. These link to form edge-sharing octahedra shown in red and that form a 3D net resulting in the **hlz** topology.

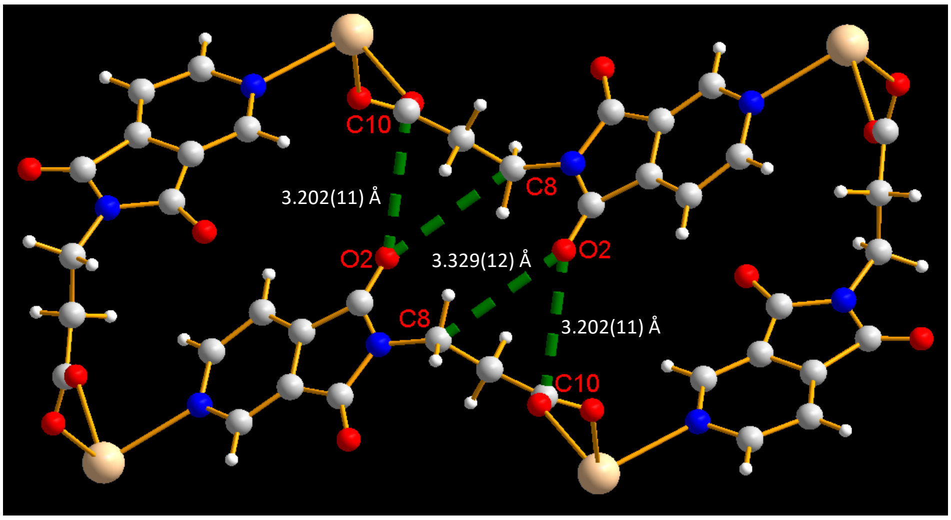

Figure S14: Select close interactions between the 3D framework in Cd(PyImPr)$_2$-**hlz**-β.

PXRD Data

Cd(PyImPr)$_2$-2D

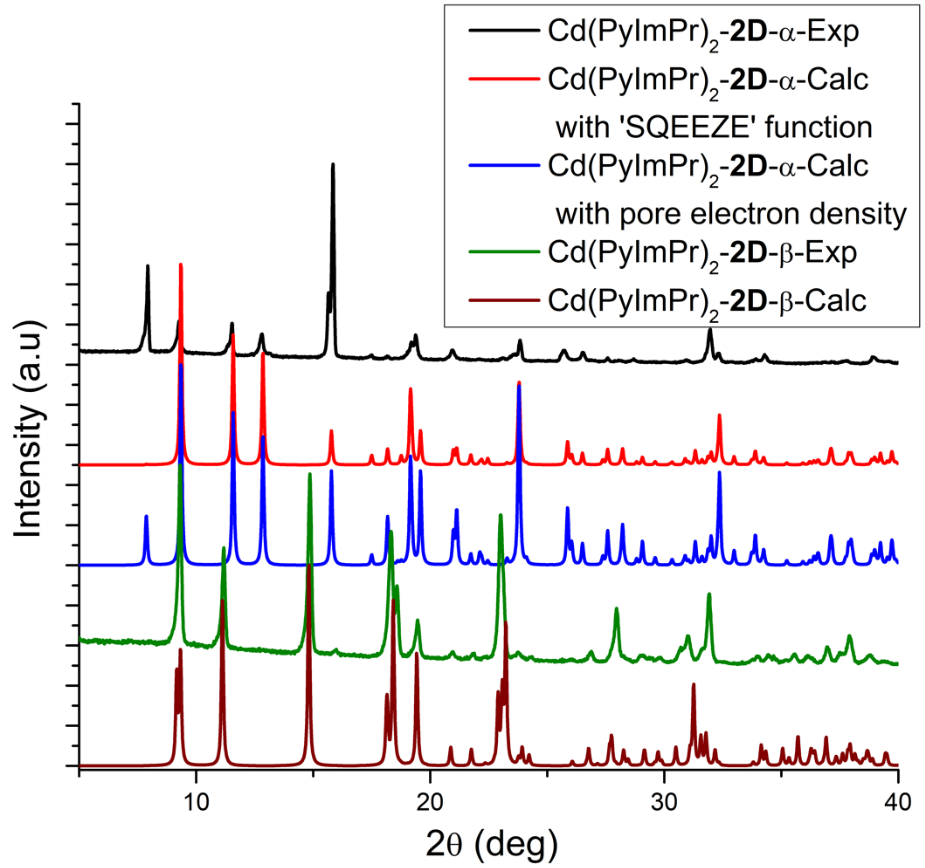

Figure S15: Overlay of experimental and calculated PXRD patterns of Cd(PyImPr)$_2$-**2D**-α and Cd(PyImPr)$_2$-**2D**-β. Calculated PXRD patterns were generated from crystals collected at 100 K while experimental patterns were collected at RT. A discrepancy is notable for Cd(PyImPr)$_2$-**2D**-α whereby the peak at 7.89° (corresponding to the 001 plane) has very low intensity in the calculated PXRD (red) pattern. This arises from the fact that the single crystal structure has the disordered solvent removed from the model by the 'SQUEEZE' function. Adding electron density to the pore results in a calculated PXRD (blue) with a larger 001 peak intensity which aligns better with the peak seen in the experimental PXRD (black).

Cd(PyImPr)$_2$-**hlz**

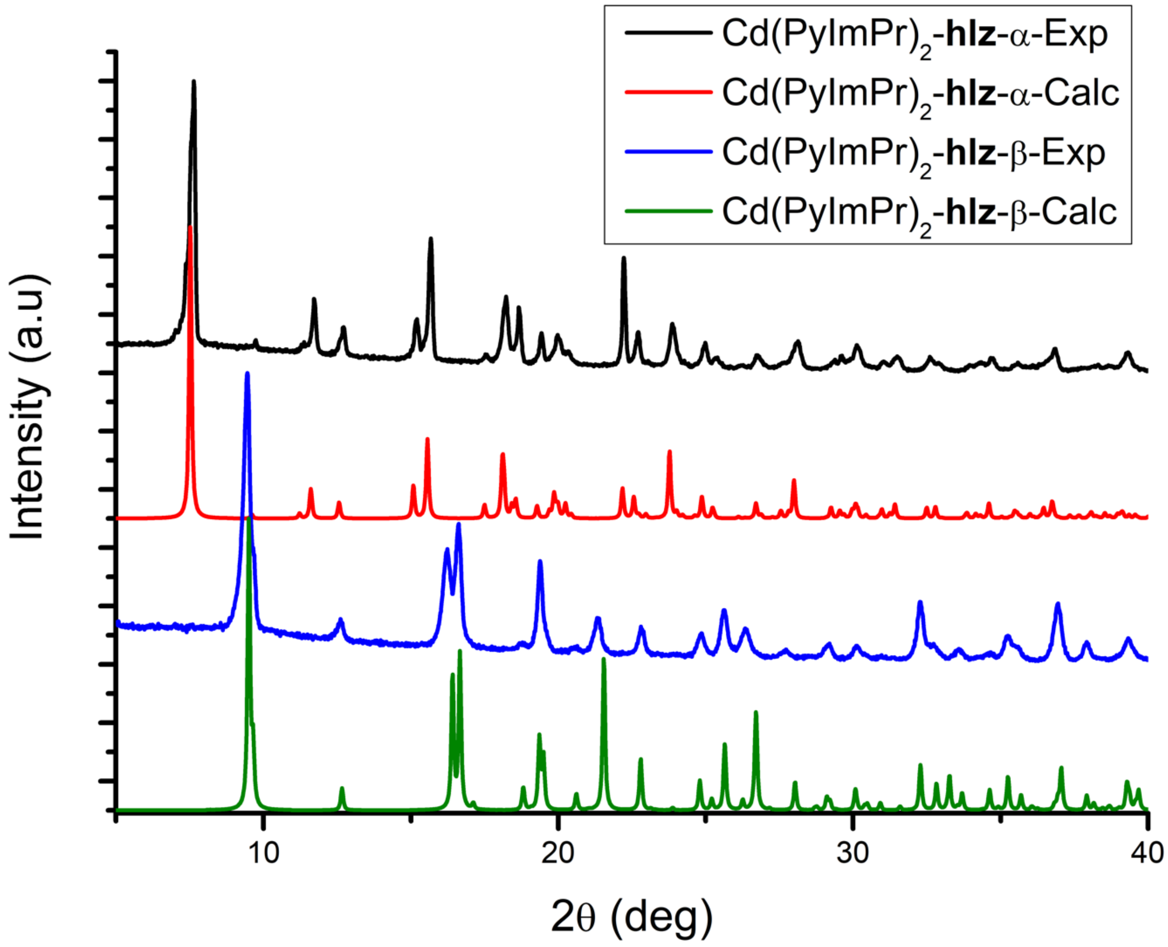

Figure S16: Overlay of experimental and calculated PXRD patterns of Cd(PyImPr)$_2$-**hlz**-α and Cd(PyImPr)$_2$-**hlz**-β. Calculated PXRD patterns were generated from crystals collected at 100 K while experimental patterns were collected at RT.

Cd(PyImPr)$_2$-**2D** soaking experiments

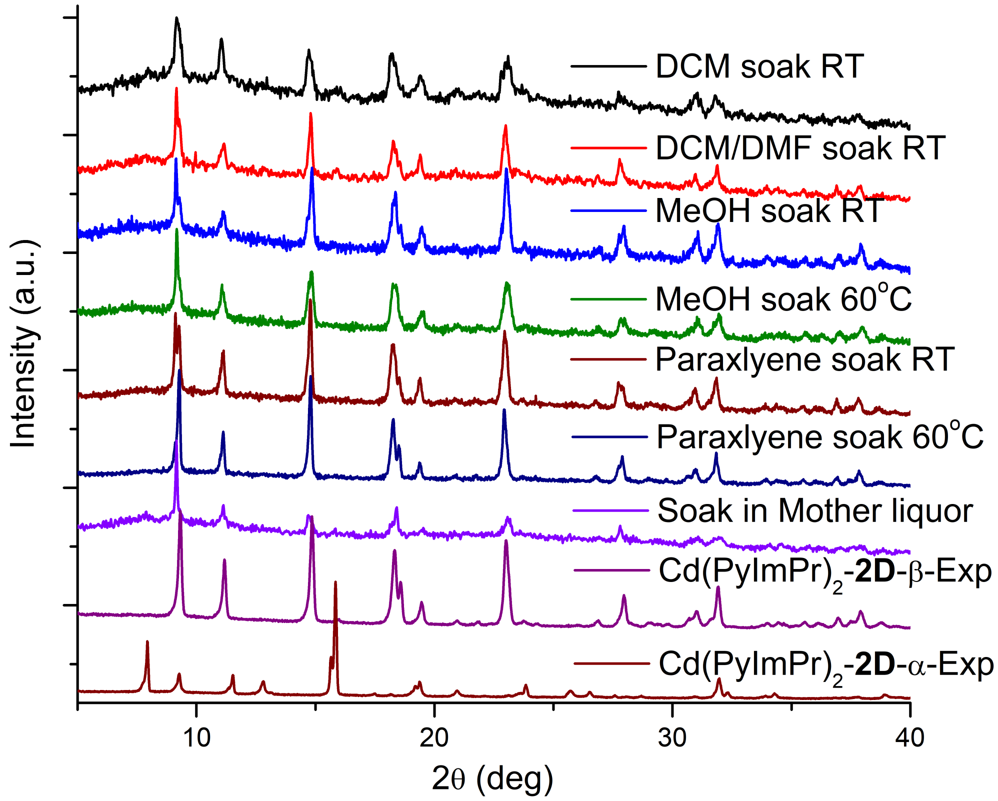

Figure S17: Overlay of experimental PXRD patterns of Cd(PyImPr)$_2$-**2D**-β after soaking in 1 ml DCM at RT for 24 h (black), 2 ml of a 1:1 mixture of DCM/DMF at RT for 24 h (red), 1 ml MeOH at RT for 24 h (blue), 1 ml MeOH at 60 °C for 24 h (green), 1 ml paraxylene at RT for 24 h (burgundy), 1 ml paraxylene at 60 °C for 24 h (navy blue), 1 ml of the mother liquor from which Cd(PyImPr)$_2$-**2D** was grown at RT for 24 h (purple) with experimental patterns of the Cd(PyImPr)$_2$-**2D**-β (pink) and Cd(PyImPr)$_2$-**2D**-α (brown).

Cd(PyImPr)$_2$-**hlz** soaking experiments

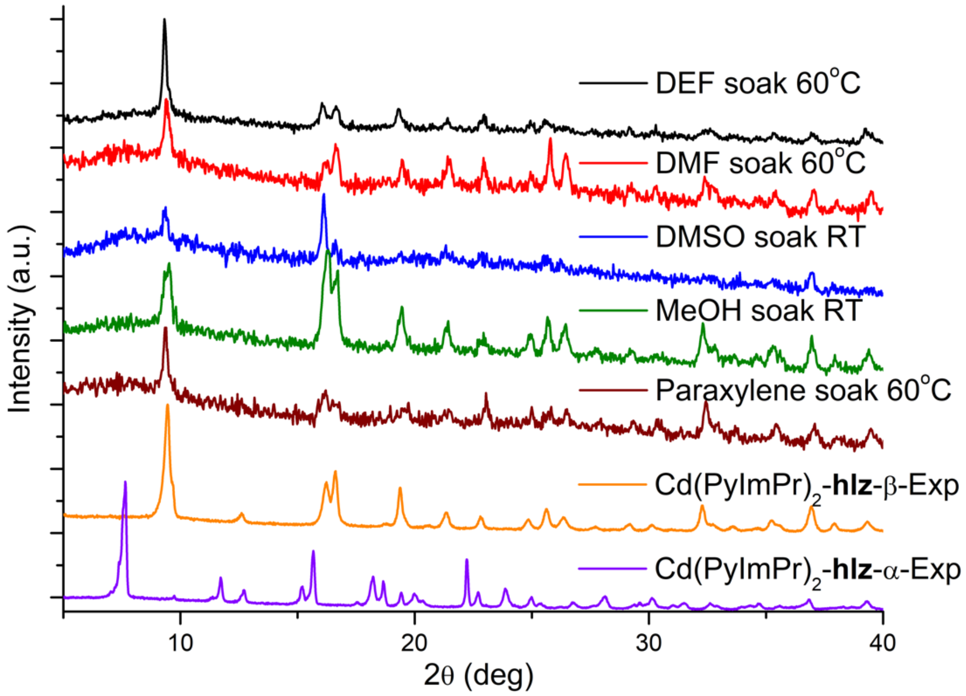

Figure S18: Overlay of experimental PXRD patterns of Cd(PyImPr)$_2$-**hlz**-β after soaking in 1 ml DEF at 60 °C for 24 h (black), 1 ml DMF at 60 °C for 24 h (red), 1 ml DMSO at RT for 24 h (blue), 1 ml MeOH at RT for 24 h (green), 1 ml paraxylene at 60 °C for 24 h (burgundy), with experimental patterns of the Cd(PyImPr)$_2$-**hlz**-β (orange) and Cd(PyImPr)$_2$-**hlz**-α (purple).

TGA Data

Cd(PyImPr)$_2$-**2D**

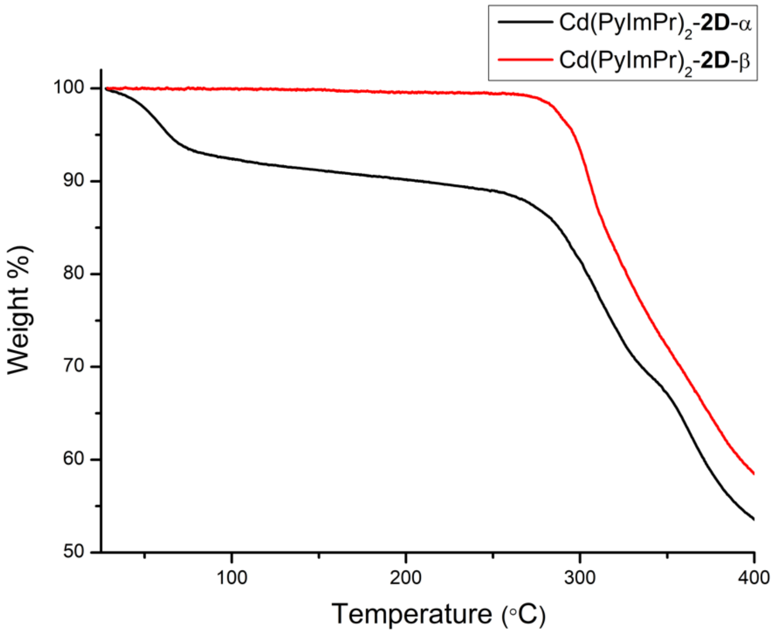

Figure S19: Overlay of TGA performed on Cd(PyImPr)$_2$-**2D**-α and Cd(PyImPr)$_2$-**2D**-β. Experimental mass loss 6.9 % at 80 °C and 11.0 % at 250 °C, calculated 11.6 % (based on Cd(PyImPr)$_2$(MeOH)$_2$).

Cd(PyImPr)$_2$-**hlz**

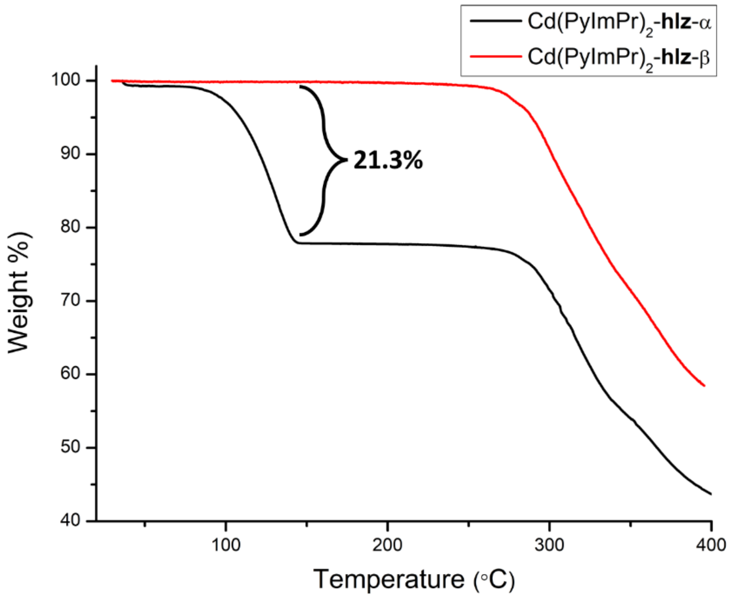

Figure S20: Overlay of TGA performed on Cd(PyImPr)$_2$-**hlz**-α and Cd(PyImPr)$_2$-**hlz**-β. Experimental mass loss 21.3 %, calculated 21.0 % (based on Cd(PyImPr)$_2$(DMF)$_2$).

DSC Data

Cd(PyImPr)$_2$-**2D**-α

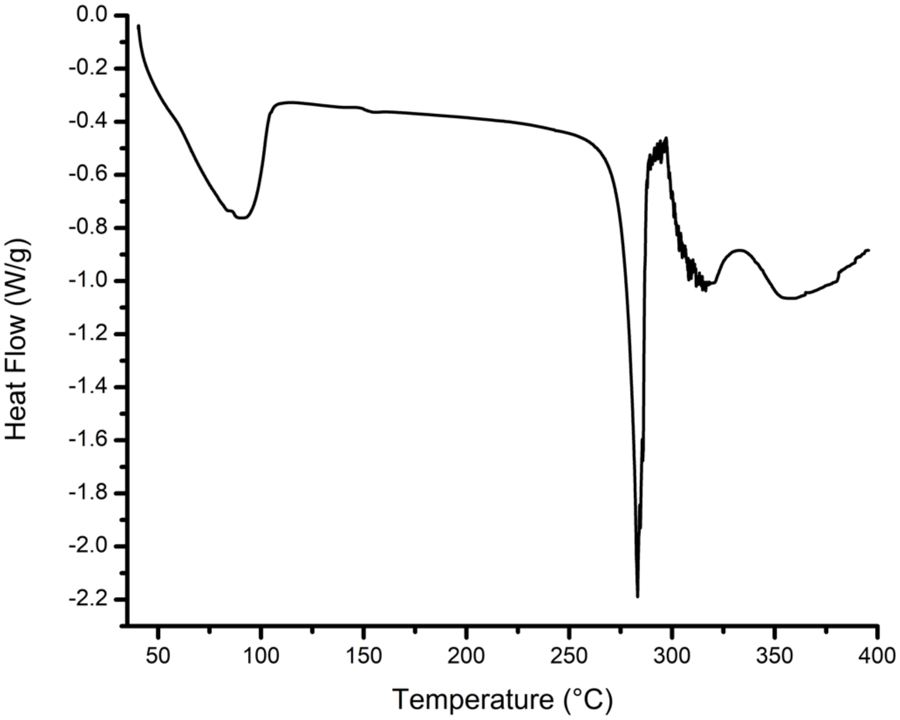

Figure S21: DSC trace of Cd(PyImPr)$_2$-**2D**-α.

Cd(PyImPr)$_2$-**2D**-β

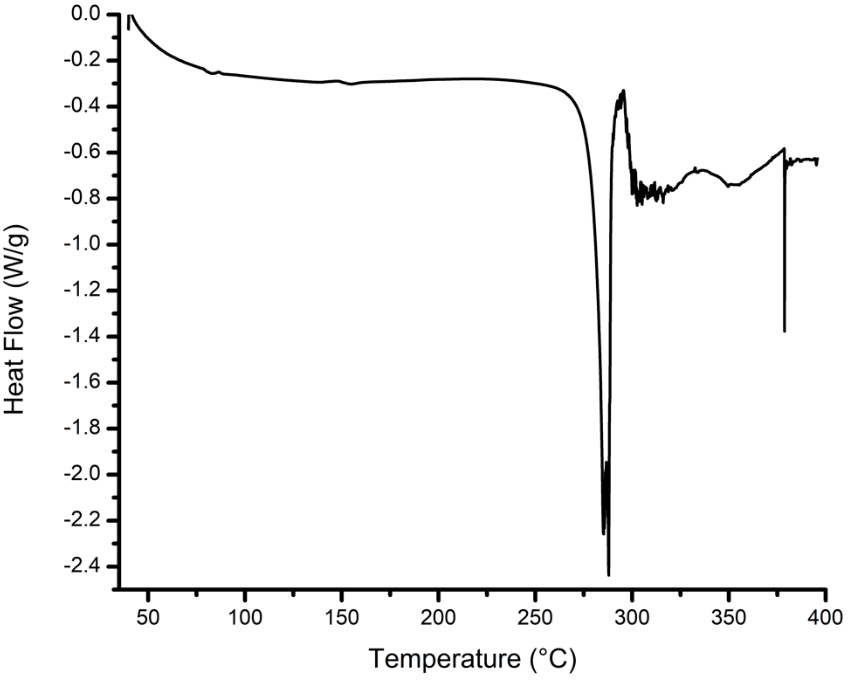

Figure S22: DSC trace of Cd(PyImPr)$_2$-**2D**-β.

Cd(PyImPr)$_2$-**hlz**-α

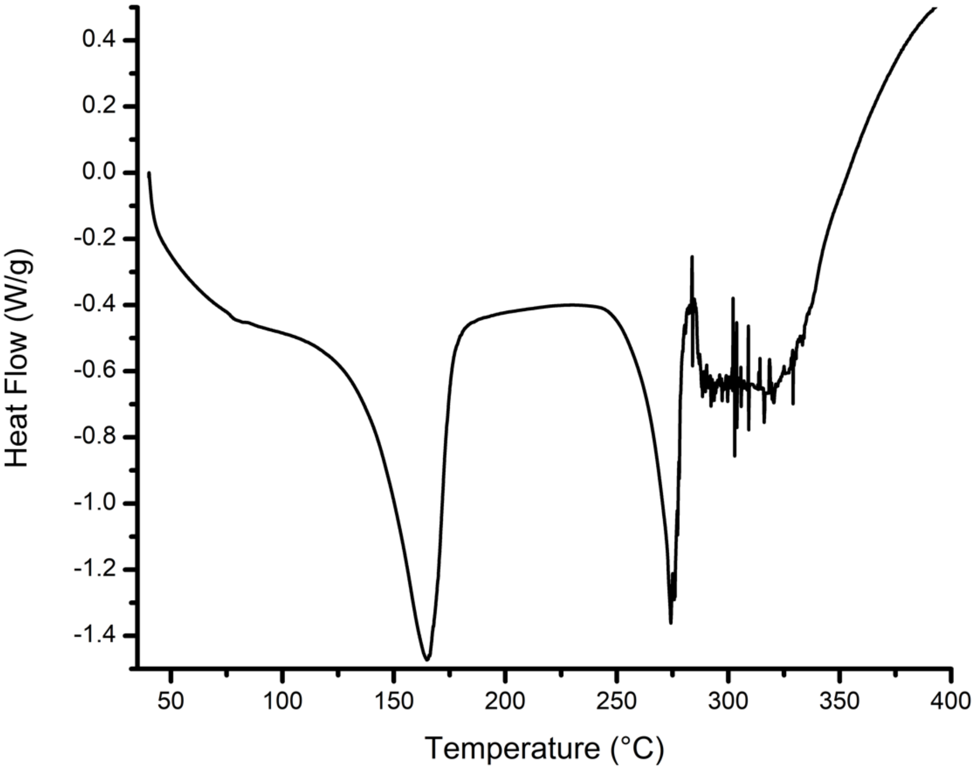

Figure S23: DSC trace of Cd(PyImPr)$_2$-**hlz**-α.

Cd(PyImPr)$_2$-**hlz**-β

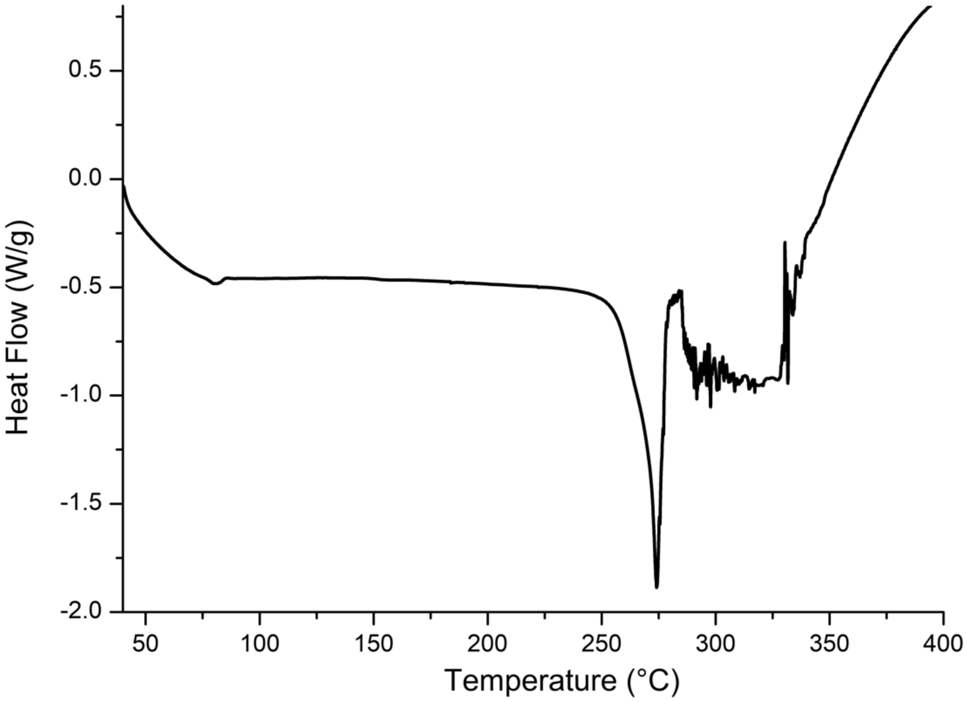

Figure S24: DSC trace of Cd(PyImPr)$_2$-**hlz**-β.

Crystallographic table

Table S1. Selected Crystallographic Data and Structure Refinement Parameters

**Compound**	**Cd(PyImPr)$_2$-2D-α**	**Cd(PyImPr)$_2$-2D-β**	**Cd(PyImPr)$_2$-hlz-α**	**Cd(PyImPr)$_2$-hlz-β**
Formula	$[C_{20}H_{14}CdN_4O_8]_n$ [+solvent]	$[C_{20}H_{14}CdN_4O_8]_n$	$[C_{20}H_{14}CdN_4O_8]_n$ $[C_3H_7NO]_{2n}$	$[C_{20}H_{14}CdN_4O_8]_n$
Wavelength (λ)	Cu K-α (1.5418 Å)	Cu K-α (1.5418 Å)	Cu K-α (1.5418 Å)	Cu K-α (1.5418 Å)
MW (g·mol^{-1})	550.75	550.75	696.94	550.75
T (K)	100	100	100	100.15
Crystal system	Triclinic	Triclinic	Monoclinic	Monoclinic
Space group	P-1	P-1	$P2_1/c$	$P2_1/c$
a (Å)	5.01730(10)	4.9864(5)	5.09680(10)	4.9218(2)
b (Å)	9.6508(3)	10.0378(11)	18.2442(3)	18.3280(7)
c (Å)	11.4756(3)	10.0982(11)	15.0256(3)	10.9159(5)
α (°)	82.3570(10)	72.982(5)	90.00	90.00
β (°)	79.5680(10)	83.424(6)	96.0580(10)	97.893(2)
γ (°)	80.0060(10)	79.174(5)	90.00	90.00
V (Å^3)	535.21(2)	473.75(9)	1389.38(5)	975.36(7)
ρ_{calc} (mg·m^{-3})	1.709	1.930	1.666	1.875
Z, Z'	1, 0.5	1, 0.5	2, 0.5	2, 0.5
Observed reflections	8611	5542	13927	14024
R_1, wR_2 [I > 2σ(I)]	0.0407, 0.1063	0.0779, 0.2047	0.0310, 0.0790	0.0757, 0.1779
R_1, wR_2 (all data)	0.0409, 0.1082	0.0815, 0.2172	0.0320, 0.0798	0.0773, 0.1785
Goodness-of-fit on F^2	1.054	1.111	1.040	1.323
R_{int} value (%)	4.96	5.53	4.97	5.34
CCDC number	2241488	2241486	2241489	2241487

Database mining

Example of shortcoming in ConQuest search function for periodic structures

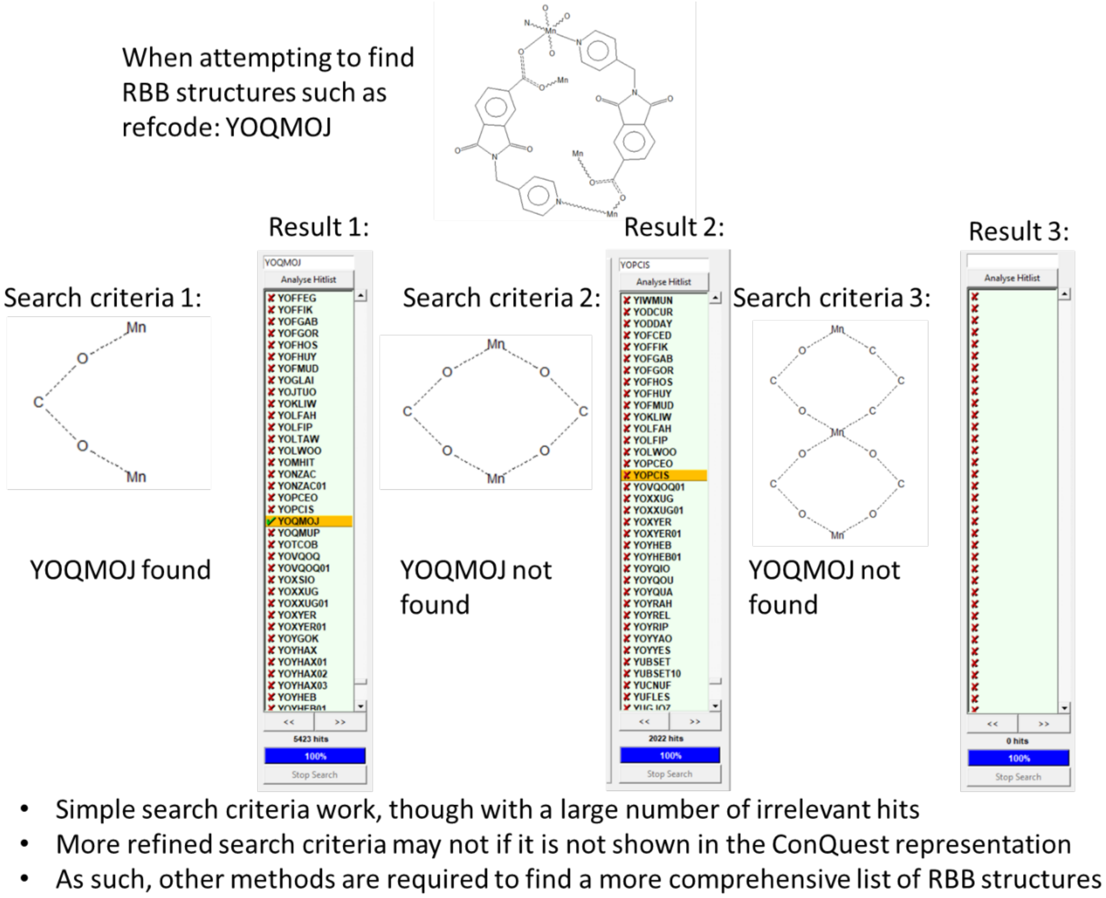

Figure S25: Example of shortcoming for ConQuest search whereby for a periodic structure, a search query must match a section of the diagrammatic representation of the structure which results in too broad a search if only a small section is used (as in search criteria 1), or the desired structure is not found at all if the search criteria goes beyond the diagrammatic representation (as in search criteria 2 and 3).

Mining Methodology

The datamining strategy used in this work to identify single-linker ML_2 MOFs (M = metal ion, L = bifunctional N-donor carboxylate ligand) and identify their topologies is comprised of 6 steps schematically illustrated in Figure S26. Each unique refcode was treated as an individual entry.

Step 1. A list of MOFs containing 116981 refcodes was obtained from the Cambridge Structural Database, CSD 'MOF subset' (version: Sept 2022).[1]

Step 2. Structures with ML_2 stoichiometry were identified using an analysis of the compound names in the list from step 1. Compound names were obtained from the CSD using the Application Programming Interface (CSD Python API).[2] Analysis of the compound names was performed using custom-written Python script which implements the workflow briefly described below. Each compound name in the CSD can be broken down to parts corresponding to the linker, metal, and solvent. A few representative examples are provided in Figure S27 to illustrate this. Stoichiometric coefficients for the linker and metal were extracted from the name text and so stoichiometric linker to metal (L/M) ratio was determined. The list from step 1 was narrowed down to single-linker structures having L/M = 2 (6943 refcodes).

We note that structures wherein the compound names in the database do not follow this naming convention were not included in these results. For example, the **hlz** structure, Mn(3-(pyridin-4-yl)acrylic acid)$_2$ (refcode: OTIQAL), has the compound name "catena-((μ-3-(pyridin-4-yl)acrylato)-manganese dimethylformamide solvate)" in the CSD giving an L/M ratio of 1, despite in-fact having 2 ligands per metal. These structures have not been included to ensure consistency.

Step 3. Structures having $M(N)_2(O)_2$ coordination were identified using the ConQuest[3] query shown in figure S28. This narrows the list from step 2 to 2980 structures.

Step 4. Structures having linkers with 'N-donor' & 'carboxylate' groups both coordinated to different metal atoms were identified to find single-linker structures with bifunctional N-donor carboxylate linkers. This analysis was performed using custom-written Python script which implements the algorithm reported by us previously.[4]

Additionally, exceptions were handled in the following way:

> Structures involving M^{3+} ions involved additional counter anions attached to the metal and were excluded: ETALUJ, LIRCUN, LOTJUB, ZECGIB, ZECHEY, ZECHIC, ZECHOI, ZECHUO.
>
> Structures based on racemate linkers were treated as single-linker ML_2 structures and so were included in the results: AVOGID, SUCMEL, WUWVOA.
>
> Due to single-crystal structure disorder in the linker, 29 structures were automatically identified by the code as being mixed-linker structures. Manual inspection confirmed these to be single-linker structures and so were included.

This resulted in 1138 structures being selected for further analysis.

Step 5. The list of refcodes from step 4 was matched with topologies reported in the TOPOS TTO database (version: Dec 2021)[5] using a custom-written Python script. Valence-bonded MOF topology determinations in either the standard or cluster representation were used. In

the rare examples where the standard representation and the cluster representation resulted in differing topologies (as in AHIFAB, ARUWUH, BEPTUM, BEQJEP, DAFZAM, ECIHOQ, HUDQUT, IWORET, MEDQOC, NUNVOI01, TUVYAL, WOFVET, XEWGEN) the standard representation was used as the “automated topology determination”.

Step 6. The list of refcodes from step 4 was manually inspected to detect RBBs and assign updated topologies.

> For structures with finite molecular building blocks (MBBs), this would typically equate to the metal centres and ligands or, where clusters were involved, points that link metal clusters together, such as the carboxylate carbons surrounding the copper paddlewheel MBB in HKUST-1.[6]
>
> In the case of RBBs, metal centres are linked through common points of extension that form the nodes for the resultant network topology.[7] For the ML_2 bifunctional N-donor carboxylates discussed here, this would typically include the carboxylate carbons and N-donors of the linker. (Figure S29)

Net simplification and topology determination is then performed on Topospro,[5] giving the final topology.

Lead coordination compounds required additional consideration. A common coordination mode of Pb^{2+} involves so-called hemi-directional[8] bonding wherein a Pb ion forms 6 shorter interactions and 2 marginally longer interactions. There is inconsistency in the literature and also in mercury as to whether the longer interactions should be considered as formal bonds (see NICOPB/NICOPB02/NICOPB04 and NICOPB01/NICOPB03). For the purposes of topology determination, all such interactions, where they are evident, are considered as formal bonds. Where this is present the row is marked with †.

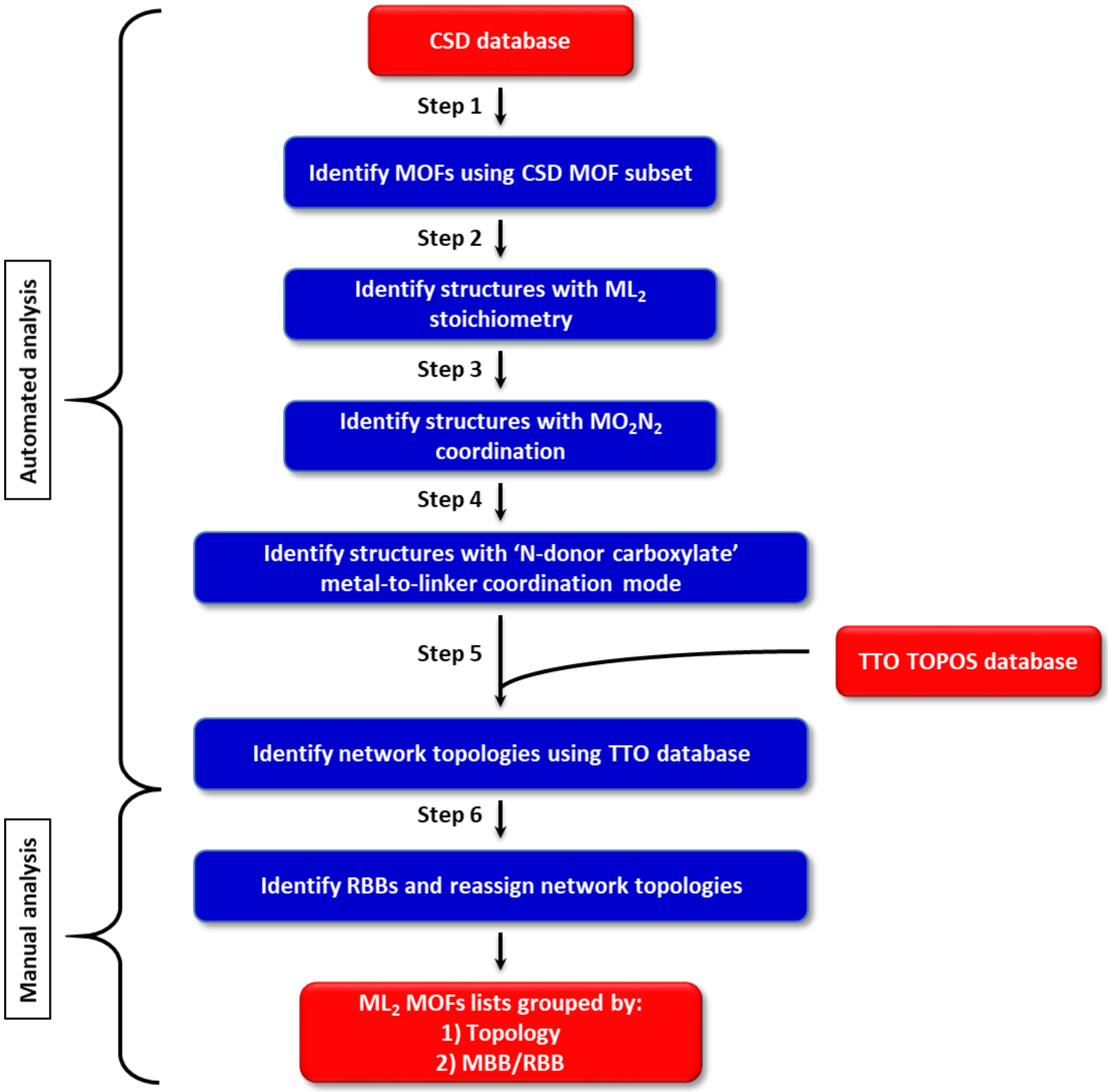

Figure S26: Database mining workflow used to identify single-linker ML_2 MOFs (M = metal ion, L = bifunctional N-donor carboxylate linker) and identify their topologies.

RefCode	Compound Name	Analysis Outcome
ACICOH	«catena-[bis(μ-isonicotinato)-nickel(ii) N,N-dimethylformamide solvate]»	L/M = 2/1 = 2
ABAVIJ	«catena-(tetrakis(m_3-isonicotinato)-di-cobalt) ethanol clathrate»	L/M = 4/2 = 2
ACOVEU	«catena-((μ4-L-Tartrato)-cobalt(ii))»	L/M = 1/1 = 1
ABAYUY	«catena-((μ2-1,2-bis(4-pyridyl)ethane)-(μ2-5-hydroxyisophthalato)-zinc(ii))»	Mixed-linker network

Figure S27: Representative examples demonstrating compound name analysis (Step 2).

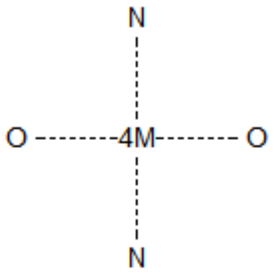

Figure S28: ConQuest query used to identify structures having $M(N)_2(O)_2$ metal coordination (Step 3).

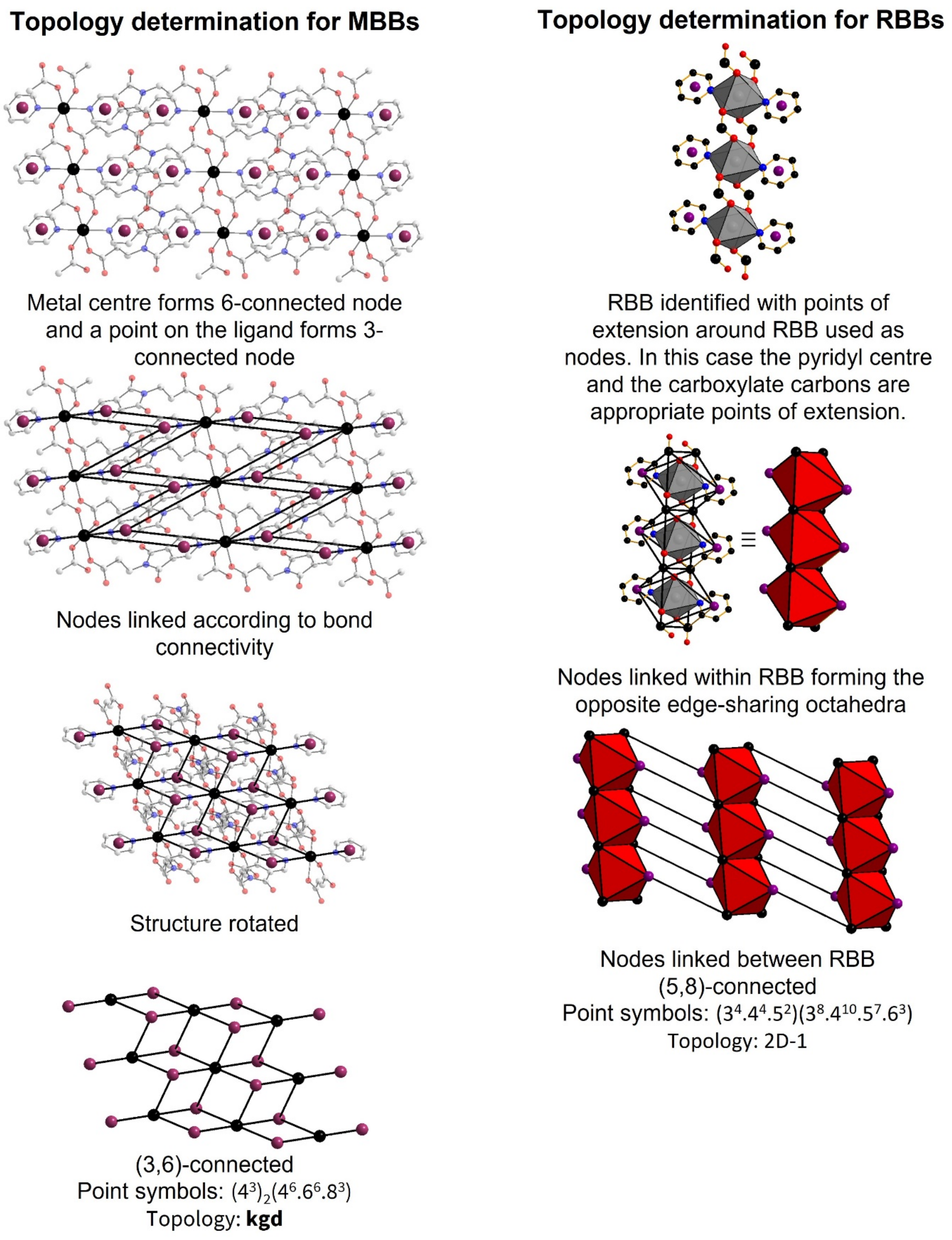

Figure S29: Example of topology determination using Cd(PyImPr)$_2$-**2D**. As can be seen with the topology determination for MBBs (left), the end result loses the information regarding the 1D chain while this information is retained on the right.

Datamining results

ML_2 structures based on N-donor carboxylate linker

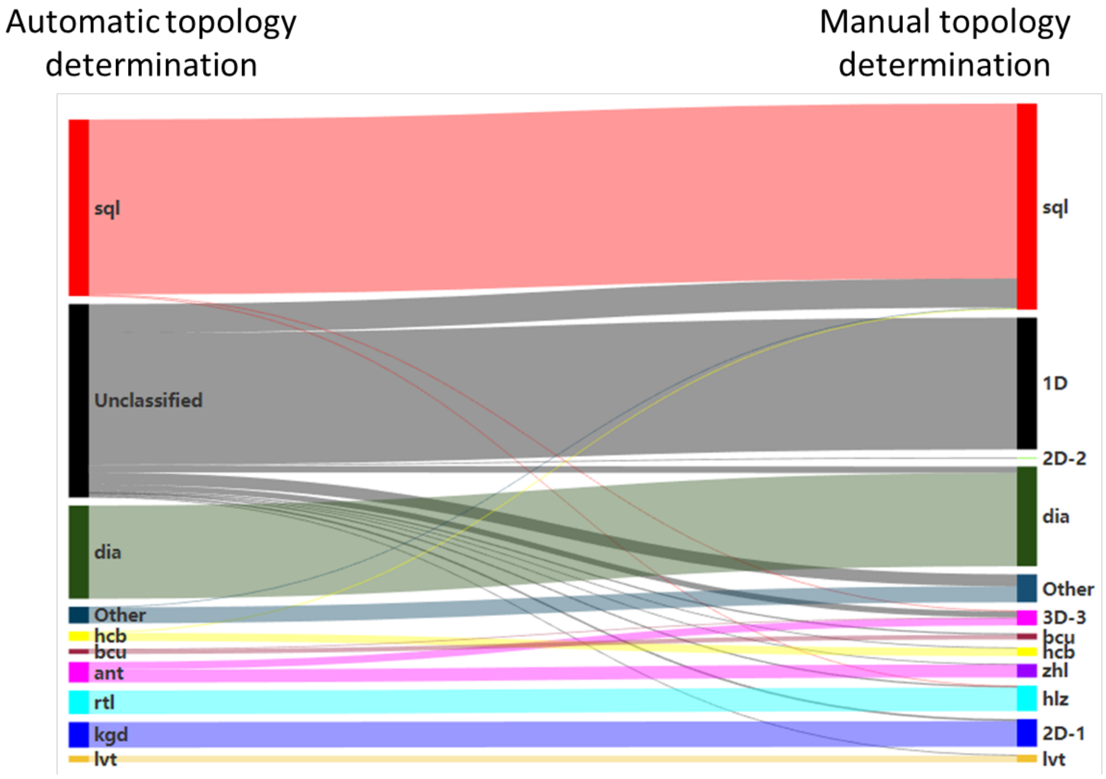

Figure S30: Sankey diagram representing the number of refcodes from each automatically determined topology (left) that result in a given manually determined topology (right).

Manual topology determination (columns); **Automatic topology determination** (rows)

	1D	sql	dia	hlz	3D-3	zhl	2D-1	2D-2	lvt	hcb	bcu	Other	Sum
Unclassified	263	58	13	3	12	1	4	2	2	1	3	24	386
sql		349		1	2								352
dia			186										186
rtl				47									47
ant					15	26							41
kgd							51						51
lvt									13				13
hcb		3								16			19
bcu					1						9		10
Other		1										32	33
Sum	263	411	199	51	30	27	55	2	15	17	12	56	1138

Figure S31: Correlation matrix representing the number of refcodes from each automatically determined topology (rows) that result in a given manually determined topology (columns).

3D N-donor carboxylate RBB topologies

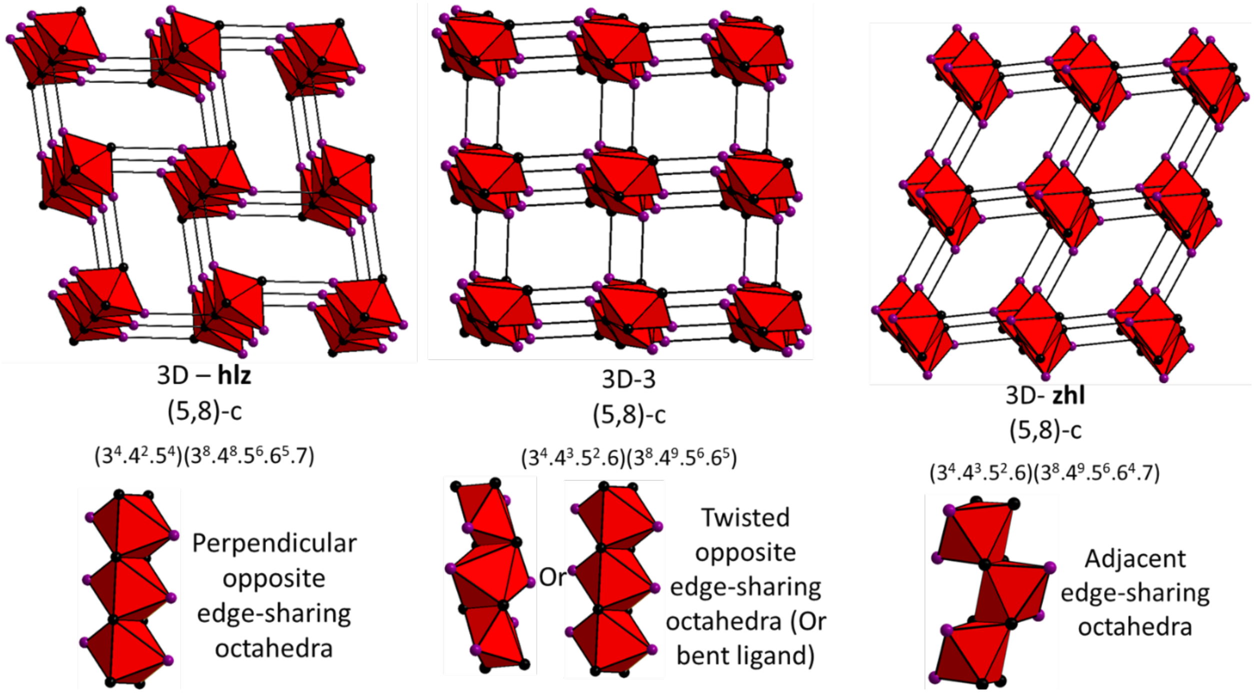

Figure S32: Possible 3D RBB topologies for N-donor carboxylates and their respective point groups (top). Points of extension for each topology are shown through carboxylate carbons (black) and the N-donors (purple). The RBB (bottom) in the **hlz** and **3D-3** topologies are based on opposite-edge sharing octahedra while the **zhl** topology is based on adjacent-edge sharing octahedra. In the case of the **3D-3** topology, network connectivity is either achieved by each edge-sharing octahedra twisting along the RBB, to facilitate bonding to alternating RBBs (see LUFCAS), or through the use of ligands with significantly bent conformations (see JOJJEY).

2D N-donor carboxylate RBB topologies

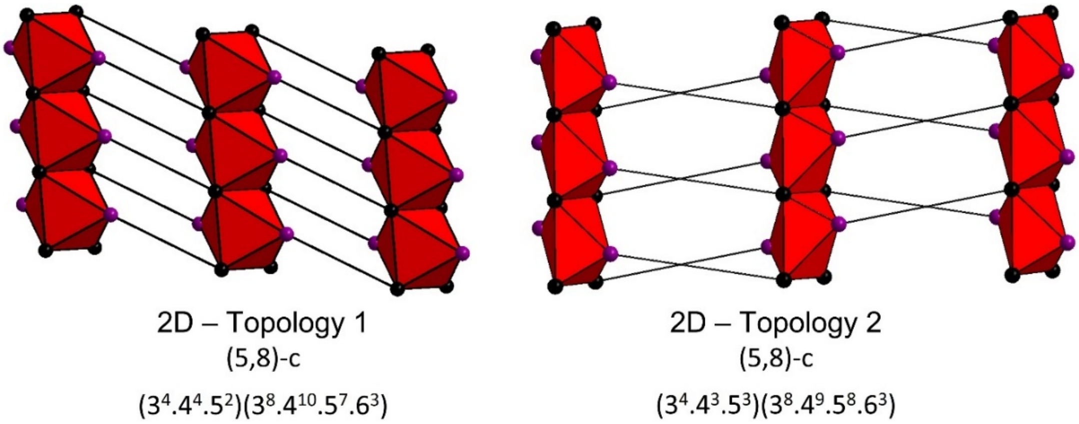

Figure S33: Possible 2D RBB topologies for N-donor carboxylates and their respective point groups. Points of extension for each topology are shown through carboxylate carbons (black) and the N-donors (purple). The RBBs in the **2D-1** and **2D-2** topologies are based on opposite-edge sharing octahedra.

ML_2 structures based on N-donor carboxylate linkers with edge-sharing octahedra RBBs

Step 6 of database mining identified 165 ML_2 refcodes based on N-donor carboxylate linkers and edge-sharing octahedra RBBs. Table S2 lists the structures with RBBs. The structures presented in this manuscript have been included at the end of the table**.**

Table S2: List of known ML_2 RBB structures made of bifunctional ligands.

Name	Network dimensionality	Topology	Point Symbol	Linker	Metal ion	CSD Refcode/ Number	Year	DOI and reference
$Fe(INA)_2$	3D	**hlz**	(5,8)-c $(3^4.4^2.5^4)(3^8.4^8.5^6.6^5.7)$	Isonicotinic acid	Fe^{2+}	HIBVET,<br>DARNUJ	1998,<br>2022	10.1039/A806499G[9]<br>10.1002/asia.202101305[10]
$Co(INA)_2$	3D	**hlz**	(5,8)-c $(3^4.4^2.5^4)(3^8.4^8.5^6.6^5.7)$	Isonicotinic acid	Co^{2+}	ABAVIJ/ABAVOP,<br>TATSIS,<br>TATSIS01,<br>CUHLID	2004,<br>2012,<br>2012,<br>2020	10.1039/b404485a[11]<br>10.1002/chem.201102295[12]<br>10.3866/PKU.WHXB201206123[13]<br>10.1016/j.ica.2020.119728[14]
$Mg(INA)_2$	3D	**hlz**	(5,8)-c $(3^4.4^2.5^4)(3^8.4^8.5^6.6^5.7)$	Isonicotinic acid	Mg^{2+}	XEQLEM,<br>XEQLEM01,<br>KOPLAF	2013,<br>2014,<br>2019	10.1039/C2DT31394D[15]<br>10.1039/C3CE42660B[16]<br>10.1039/C9MH00133F[17]
$Mn(INA)_2$	3D	**hlz**	(5,8)-c $(3^4.4^2.5^4)(3^8.4^8.5^6.6^5.7)$	Isonicotinic acid	Mn^{2+}	HIVQAG,<br>OTIQEP,<br>IVELUS/IVEMAZ/IVEMED/IVEMIH/IVEMIH01,<br>FEJFAE,<br>NAHBOR/NAGZAA/IVEMIH02/IVEMIH03/IVEMIH04	2014,<br>2016,<br>2016,<br><br>2017,<br>2020	10.1039/c3ce42660b[16]<br>10.1021/acs.inorgchem.6b00758[18]<br>10.1002/chem.201601784[19]<br><br>10.1039/C7CE01766A[20]<br>10.1021/jacs.0c09475[21]
$Cd(INA)_2$	3D	**hlz**	(5,8)-c $(3^4.4^2.5^4)(3^8.4^8.5^6.6^5.7)$	Isonicotinic acid	Cd^{2+}	GIMSUS,<br>SIVVAW	2013,<br>2014	10.7868/S0132344X13030080[22]<br>10.1039/C3CE42216J[23]
Mn-FINA-1	3D	**hlz**	(5,8)-c $(3^4.4^2.5^4)(3^8.4^8.5^6.6^5.7)$	3-fluoroisonicotinic acid	Mn^{2+}	OTIPOY	2016	10.1021/acs.inorgchem.6b00758[18]
Cd-FINA-1	3D	**hlz**	(5,8)-c $(3^4.4^2.5^4)(3^8.4^8.5^6.6^5.7)$	3-fluoroisonicotinic acid	Cd^{2+}	OTOVEA	2016	10.1021/acs.inorgchem.6b00758[18]
Co-FINA-1	3D	**hlz**	(5,8)-c $(3^4.4^2.5^4)(3^8.4^8.5^6.6^5.7)$	3-fluoroisonicotinic acid	Co^{2+}	TATSEO	2012	10.1002/chem.201102295[12]
Mn-FPAA-1	3D	**hlz**	(5,8)-c $(3^4.4^2.5^4)(3^8.4^8.5^6.6^5.7)$	3-(3-fluoropyridin-4-yl)acrylic acid	Mn^{2+}	OTIPUE	2016	10.1021/acs.inorgchem.6b00758[18]
Co-FPAA-1	3D	**hlz**	(5,8)-c $(3^4.4^2.5^4)(3^8.4^8.5^6.6^5.7)$	3-(3-fluoropyridin-4-yl)acrylic acid	Co^{2+}	OTOVIE	2016	10.1021/acs.inorgchem.6b00758[18]
Cd-FPAA-1	3D	**hlz**	(5,8)-c $(3^4.4^2.5^4)(3^8.4^8.5^6.6^5.7)$	3-(3-fluoropyridin-4-yl)acrylic acid	Cd^{2+}	OTOVAW	2016	10.1021/acs.inorgchem.6b00758[18]
$Cd(tetza)_2$	3D	**hlz**	(5,8)-c $(3^4.4^2.5^4)(3^8.4^8.5^6.6^5.7)$	1H-tetrazol-1-ylacetic acid	Cd^{2+}	JOJHUM	2008	10.1021/cg7012182[24]
$Mn(tetza)_2$	3D	**hlz**	(5,8)-c $(3^4.4^2.5^4)(3^8.4^8.5^6.6^5.7)$	1H-tetrazol-1-ylacetic acid	Mn^{2+}	JOJJAU	2008	10.1021/cg7012182[24]
$Co(tetza)_2$	3D	**hlz**	(5,8)-c $(3^4.4^2.5^4)(3^8.4^8.5^6.6^5.7)$	1H-tetrazol-1-ylacetic acid	Co^{2+}	JOJJIC	2008	10.1021/cg7012182[24]
$Cu(tetza)_2$	3D	**hlz**	(5,8)-c $(3^4.4^2.5^4)(3^8.4^8.5^6.6^5.7)$	1H-tetrazol-1-ylacetic acid	Cu^{2+}	KOBGOY02	2009	10.1016/j.ica.2008.10.009[25]
$Mn(CIP)_2$	3D	**hlz**	(5,8)-c $(3^4.4^2.5^4)(3^8.4^8.5^6.6^5.7)$	4-(4-carboxylphenyl)-2,6-di(4-imidazol-1-yl)phenyl)pyridine)	Mn^{2+}	SOFWOC	2019	10.1039/C9DT01430F[26]
$Co(L1')_2$	3D	**hlz**	(5,8)-c $(3^4.4^2.5^4)(3^8.4^8.5^6.6^5.7)$	(3-cyanophenyl)-[2,3'-bipyridin]-6-yl)benzoic acid	Co^{2+}	LAJKIU	2020	10.1016/j.poly.2020.114918[27]
$Mn(L1')_2$	3D	**hlz**	(5,8)-c $(3^4.4^2.5^4)(3^8.4^8.5^6.6^5.7)$	(3-cyanophenyl)-[2,3'-bipyridin]-6-yl)benzoic acid	Mn^{2+}	LAJKOA	2020	10.1016/j.poly.2020.114918[27]
$Co(IMPAA)_2$	3D	**hlz**	(5,8)-c $(3^4.4^2.5^4)(3^8.4^8.5^6.6^5.7)$	(2E)-3-[4-(1H-imidazol-1-ylmethyl)phenyl]acrylic acid	Co^{2+}	DUFCEO	2015	10.14102/j.cnki.0254-5861.2011-0419[28]

Cd(3-pyoa)$_2$	3D	**hlz**	(5,8)-c $(3^4.4^2.5^4)(3^8.4^8.5^6.6^5.7)$	5-(3-pyridyl)-1,3,4-oxadiazole-2-thioacetic acid	Cd^{2+}	KOHBUF	2008	10.1039/b803736a[29]
Cd(4-tzbc)$_2$	3D	**hlz**	(5,8)-c $(3^4.4^2.5^4)(3^8.4^8.5^6.6^5.7)$	4-(1,2,4-triazolylmethyl) benzoic acid	Cd^{2+}	BEZKOI,<br>BEZKOI01	2012,<br>2015	10.1016/j.synthmet.2012.07.024[30]<br>10.1039/C4TA06671E[31]
Co(imta)2**	3D	**hlz**	(5,8)-c $(3^4.4^2.5^4)(3^8.4^8.5^6.6^5.7)$	2-{4-[(1H-imidazol-1-yl)methyl]-1H-1,2,3-triazol-1-yl}acetic acid	Co^{2+}	NAFTIA	2016	10.1039/C5CE02277K[32]
Mn(imta)2	3D	**hlz**	(5,8)-c $(3^4.4^2.5^4)(3^8.4^8.5^6.6^5.7)$	2-{4-[(1H-imidazol-1-yl)methyl]-1H-1,2,3-triazol-1-yl}acetic acid	Mn^{2+}	NAFTOG	2016	10.1039/C5CE02277K[32]
Cd(imta)2	3D	**hlz**	(5,8)-c $(3^4.4^2.5^4)(3^8.4^8.5^6.6^5.7)$	2-{4-[(1H-imidazol-1-yl)methyl]-1H-1,2,3-triazol-1-yl}acetic acid	Cd^{2+}	NAFTUM	2016	10.1039/C5CE02277K[32]
CuL$_2$	3D	**hlz**	(5,8)-c $(3^4.4^2.5^4)(3^8.4^8.5^6.6^5.7)$	3-bromo-5-(4-carboxyphenyl)pyridine	Cu^{2+}	MODZAJ	2019	10.1016/j.molstruc.2019.02.046[33]
Cd(3-(4-(2-pyridin-3-yl-vinyl)phenyl) propenate)$_2$	3D	**hlz**	(5,8)-c $(3^4.4^2.5^4)(3^8.4^8.5^6.6^5.7)$	3-{4-(2-pyridin-3-yl-vinyl)-phenyl}-propenoic acid	Cd^{2+}	TIKWIU	2007	10.1021/cg060677j[34]
Cd(S-3-pytzipa)(R-3-pytzipa)*	3D	**hlz**	(5,8)-c $(3^4.4^2.5^4)(3^8.4^8.5^6.6^5.7)$	(S/R)5-(n-pyridyl)tetrazole-2-isopropionic acid	Cd^{2+}	SUCMEL	2017	10.1007/s10904-017-0623-8[35]
Cd(S-pztza)(R-pztza)*	3D	**hlz**	(5,8)-c $(3^4.4^2.5^4)(3^8.4^8.5^6.6^5.7)$	(S/R)5-(2-pyrazinyl)tetrazole-2(1-methyl)acetic acid	Cd^{2+}	AVOGID	2016	10.1016/j.jssc.2016.09.021[36]
MCF-44	3D	**hlz**	(5,8)-c $(3^4.4^2.5^4)(3^8.4^8.5^6.6^5.7)$	4-(4-pyridinyl)benzoic acid	Mn^{2+}	QOQWOK/QOQWOK01	2014	10.1007/s11426-013-5048-9[37]
MCF-43	3D	**zhl**	(5,8)-c $(3^4.4^3.5^2.6)(3^8.4^9.5^6.6^4.7)$	4-(3-pyridinyl)benzoic acid	Mn^{2+}	LUXBAI	2010	10.1002/zaac.200900412[38]
Mg(4,3-pyb)$_2$	3D	**zhl**	(5,8)-c $(3^4.4^3.5^2.6)(3^8.4^9.5^6.6^4.7)$	4-(3-pyridinyl)benzoic acid	Mg^{2+}	KUBXUC	2014	10.1016/j.inoche.2014.08.034[39]
MCF-34	3D	**zhl**	(5,8)-c $(3^4.4^3.5^2.6)(3^8.4^9.5^6.6^4.7)$	3-(pyridin-4-yl)benzoic acid	Mn^{2+}	PIHJOH,<br>LIJNOJ/LIJNOJ01/LIJPAX/<br>LIJPAX01/LIJPAX02/LIJPOL/<br>PIHJOH01/PIHJOH02/ PIHJOH03,<br>BOYTEQ	2013,<br>2013,<br><br><br>2014	10.1080/15533174.2012.758153[40]<br>10.1038/ncomms3534[41]<br><br><br>10.14102/j.cnki.0254-5861.2011-0319[42]
Mg(3,4-pyb)$_2$	3D	**zhl**	(5,8)-c $(3^4.4^3.5^2.6)(3^8.4^9.5^6.6^4.7)$	3-(pyridin-4-yl)benzoic acid	Mg^{2+}	KUBXOW	2014	10.1016/j.inoche.2014.08.034[39]
Mn(pmba)$_2$	3D	**zhl**	(5,8)-c $(3^4.4^3.5^2.6)(3^8.4^9.5^6.6^4.7)$	3-(pyridin-4-yl)-3-methyl-benzoic acid	Mn^{2+}	NATRUX	2012	10.1007/s10870-011-0225-1[43]
MnL$_2$	3D	**zhl**	(5,8)-c $(3^4.4^3.5^2.6)(3^8.4^9.5^6.6^4.7)$	4-(pyridin-3-ylethynyl)benzoic acid	Mn^{2+}	WOHYUN	2000	10.1021/ic000227v[44]
Mn(mpba)$_2$	3D	**zhl**	(5,8)-c $(3^4.4^3.5^2.6)(3^8.4^9.5^6.6^4.7)$	3-(3-methylpyridin-4-yl)benzoic acid	Mn^{2+}	HULGAZ/HULGED	2020	10.1021/acs.inorgchem.0c00022[45]
Co(mpba)$_2$	3D	**zhl**	(5,8)-c $(3^4.4^3.5^2.6)(3^8.4^9.5^6.6^4.7)$	3-(3-methylpyridin-4-yl)benzoic acid	Co^{2+}	HULGIH/HULGON	2020	10.1021/acs.inorgchem.0c00022[45]
Mg(NA)$_2$	3D	**zhl**	(5,8)-c $(3^4.4^3.5^2.6)(3^8.4^9.5^6.6^4.7)$	Nicotinic acid	Mg^{2+}	XEQLIQ	2013	10.1039/C2DT31394D[15]
Mn(NA)$_2$	3D	**zhl**	(5,8)-c $(3^4.4^3.5^2.6)(3^8.4^9.5^6.6^4.7)$	Nicotinic acid	Mn^{2+}	WOHYOH/WOHYOH01	2000	10.1021/ic000227v[44]
Cd(FNA)$_2$	3D	**zhl**	(5,8)-c $(3^4.4^3.5^2.6)(3^8.4^9.5^6.6^4.7)$	5-fluoronicotinic acid	Cd^{2+}	NIMVUC	2013	10.1039/C3CE41116H[46]
Mn(3-pyoa)$_2$	3D	**zhl**	(5,8)-c $(3^4.4^3.5^2.6)(3^8.4^9.5^6.6^4.7)$	5-(3-pyridyl)-1,3,4-oxadiazole-2-thioacetic acid	Mn^{2+}	DAMVEW	2017	10.17344/acsi.2016.3109[47]
Mn(HL)$_2$	3D	**zhl**	(5,8)-c $(3^4.4^3.5^2.6)(3^8.4^9.5^6.6^4.7)$	5-(4H-1,2,4-Triazol-4-yl)benzene-1,3-dicarboxylic Acid	Mn^{2+}	IWEPUW	2011	10.1021/cg200068v[48]
Cd(L^2)$_2$	3D	**zhl**	(5,8)-c $(3^4.4^3.5^2.6)(3^8.4^9.5^6.6^4.7)$	(E)-6-((pyridine-3-ylmethylene)amino)-2-napthoic acid	Cd^{2+}	REVGUW	2013	10.1016/j.poly.2013.01.019[49]
Pb(NA)$_2$**†**	3D	**3D – Topology 3**	(5,8)-c $(3^4.4^3.5^2.6)(3^8.4^9.5^6.6^5)$	Nicotinic acid	Pb^{2+}	NICOPB,<br>NICOPB01,<br>NICOPB02,<br>NICOPB03,<br>NICOPB04	1975,<br>2009,<br>2011,<br>2011,<br>2018	[No DOI Found][50]<br>10.5517/ccqpb2s[51]<br>10.1016/j.ultsonch.2010.01.011[52]<br>10.1007/s10904-011-9504-8[53]<br>10.1016/j.solidstatesciences.2018.03.004[54]
Pb(ANA)$_2$**†**	3D	**3D – Topology 3**	(5,8)-c $(3^4.4^3.5^2.6)(3^8.4^9.5^6.6^5)$	2-aminonicotinic acid	Pb^{2+}	POQLUF	2019	10.1039/C9DT02928A[55]

$Pb(FNA)_2$†	3D	**3D – Topology 3**	(5,8)-c<br>$(3^4.4^3.5^2.6)(3^8.4^9.5^6.6^5)$	5-fluoronicotinic acid	Pb^{2+}	TUCZAV	2020	10.1071/CH19416[56]
$Pb(3\text{-}pyb)_2$†	3D	**3D – Topology 3**	(5,8)-c<br>$(3^4.4^3.5^2.6)(3^8.4^9.5^6.6^5)$	3-(3-pyridyl)acrylic acid	Pb^{2+}	FEZVUC	2005	10.1002/ejic.200400648[57]
$Zn(tetza)_2$	3D	**3D – Topology 3**	(5,8)-c<br>$(3^4.4^3.5^2.6)(3^8.4^9.5^6.6^5)$	1H-tetrazol-1-ylacetic acid	Zn^{2+}	JOJJEY	2008	10.1021/cg7012182 [24]
$Cd(tetza)_2$	3D	**3D – Topology 3**	(5,8)-c<br>$(3^4.4^3.5^2.6)(3^8.4^9.5^6.6^5)$	1H-tetrazol-1-ylacetic acid	Cd^{2+}	JOJHUM01,<br>JOJHUM02	2009,<br>2015	10.1107/S160053680904255X[58]<br>10.1016/j.jssc.2015.02.024[59]
$Mn(tetza)_2$	3D	**3D – Topology 3**	(5,8)-c<br>$(3^4.4^3.5^2.6)(3^8.4^9.5^6.6^5)$	1H-tetrazol-1-ylacetic acid	Mn^{2+}	JOJJAU01	2017	10.1039/C7RA07997D[60]
$Co(tetza)_2$	3D	**3D – Topology 3**	(5,8)-c<br>$(3^4.4^3.5^2.6)(3^8.4^9.5^6.6^5)$	1H-tetrazol-1-ylacetic acid	Co^{2+}	JOJJIC01,<br>JOJJIC02	2015,<br>2017	10.1016/j.jssc.2015.08.049[61]<br>10.1039/C7RA07997D[60]
$Ni(tetza)_2$	3D	**3D – Topology 3**	(5,8)-c<br>$(3^4.4^3.5^2.6)(3^8.4^9.5^6.6^5)$	1H-tetrazol-1-ylacetic acid	Ni^{2+}	TOHRUF,<br>TOHRUF01	2014,<br>2017	10.1016/j.inoche.2014.05.039[62]<br>10.1039/C7RA07997D[60]
$Pb(tetza)_2$†	3D	**3D – Topology 3**	(5,8)-c<br>$(3^4.4^3.5^2.6)(3^8.4^9.5^6.6^5)$	1H-tetrazol-1-ylacetic acid	Pb^{2+}	IZEBUL,<br>IZEBUL01	2011,<br>2011	10.5517/ccvt4wn[63]<br>10.5517/ccv8kn9[64]
$Cd(TBA)_2$	3D	**3D – Topology 3**	(5,8)-c<br>$(3^4.4^3.5^2.6)(3^8.4^9.5^6.6^5)$	4-(1H-1,2,4-triazol-1-yl)benzoic acid	Cd^{2+}	LUFCAS	2012	10.4236/ojic.2012.23009[65]
$Pb(imta)_2$†	3D	**3D – Topology 3**	(5,8)-c<br>$(3^4.4^3.5^2.6)(3^8.4^9.5^6.6^5)$	2-{4-[(1H-imidazol-1-yl)methyl]-1H-1,2,3-triazol-1-yl}acetic acid	Pb^{2+}	NAFWOJ	2016	10.1039/c5ce02277k[32]
$Cd_2(HL)_4$	3D	**3D – Topology 3**	(5,8)-c<br>$(3^4.4^3.5^2.6)(3^8.4^9.5^6.6^5)$	1H-indazole-6-carboxylic acid	Cd^{2+}	UQIXEA	2021	10.3390/inorganics9030020[66]
$Zn(pytza)_2$	3D	**3D – Topology 3**	(5,8)-c<br>$(3^4.4^3.5^2.6)(3^8.4^9.5^6.6^5)$	5-(4-pyridyl)tetrazole-2-acetic acid	Zn^{2+}	LUFHUR	2015	10.1039/C5RA03848K[67]
$Mg(pytza)_2$	3D	**3D – Topology 3**	(5,8)-c<br>$(3^4.4^3.5^2.6)(3^8.4^9.5^6.6^5)$	5-(4-pyridyl)tetrazole-2-acetic acid	Mg^{2+}	OPIRIQ	2016	10.1016/j.ica.2016.06.015[68]
$Ni(pytza)_2$	3D	**3D – Topology 3**	(5,8)-c<br>$(3^4.4^3.5^2.6)(3^8.4^9.5^6.6^5)$	5-(4-pyridyl)tetrazole-2-acetic acid	Ni^{2+}	OZONIC	2016	10.1016/j.inoche.2016.10.007[69]
$Mn(pytza)_2$	3D	**3D – Topology 3**	(5,8)-c<br>$(3^4.4^3.5^2.6)(3^8.4^9.5^6.6^5)$	5-(4-pyridyl)tetrazole-2-acetic acid	Mn^{2+}	WAGKIB	2016	10.1007/s11243-015-0003-6[70]
Pb(4,3-<br>$pyb)_2$†	3D	**3D – Topology 3**	(5,8)-c<br>$(3^4.4^3.5^2.6)(3^8.4^9.5^6.6^5)$	4-(3-pyridinyl)benzoic acid	Pb^{2+}	GURYAU,<br>GURYAU01	2009,<br>2011	10.1016/j.jssc.2009.09.004[71]<br>10.5517/ccwg3s6[72]
$Pb(bmzbc)_2$†	3D	**3D – Topology 3**	(5,8)-c<br>$(3^4.4^3.5^2.6)(3^8.4^9.5^6.6^5)$	4-(benzimidazole-1-yl)benzoic acid	Pb^{2+}	YUDRIA	2016	10.14102/j.cnki.0254-5861.2011-0886[73]
$Pb(qlc)_2$†	3D	**3D – Topology 3**	(5,8)-c<br>$(3^4.4^3.5^2.6)(3^8.4^9.5^6.6^5)$	Quinoline-6-carboxylic acid	Pb^{2+}	BOPVIM	2009	10.1016/j.molstruc.2008.12.059[74]
$Pb(4\text{-}pyoa)_2$†	3D	**3D – Topology 3**	(5,8)-c<br>$(3^4.4^3.5^2.6)(3^8.4^9.5^6.6^5)$	5-(4-pyridyl)-1,3,4-oxadiazole-2-thioacetic acid	Pb^{2+}	KOHBIT	2008	10.1039/b803736a[29]
$Cd(PAA)_2$	2D	**2D – Topology 1**	(5,8)-c<br>$(3^4.4^4.5^2)(3^8.4^{10}.5^7.6^3)$	3-(pyridin-3-yl)acrylic acid	Cd^{2+}	FIPFIU,<br>FIPFIU01,<br>FIPFIU02,<br>FIPFIU03	2005,<br>2004,<br>2004,<br>2009	10.1002/aoc.699[75]<br>[No DOI Found][76]<br>10.5517/ccp5bkq[77]<br>10.1016/j.molstruc.2008.10.046[78]
$Mn(PAA)_2$	2D	**2D – Topology 1**	(5,8)-c<br>$(3^4.4^4.5^2)(3^8.4^{10}.5^7.6^3)$	3-(pyridin-3-yl)acrylic acid	Mn^{2+}	BIVXIO	2008	10.1039/B715460G[79]
$Cd(PPA)_2$	2D	**2D – Topology 1**	(5,8)-c<br>$(3^4.4^4.5^2)(3^8.4^{10}.5^7.6^3)$	3-pyridine propionic acid	Cd^{2+}	QATROU	2012	10.1016/j.inoche.2011.10.010[80]
$Pb(Pyta)_2$†	2D	**2D – Topology 1**	(5,8)-c<br>$(3^4.4^4.5^2)(3^8.4^{10}.5^7.6^3)$	4-pyridylthioacetic acid	Pb^{2+}	DAFZIU	2004	10.1039/b403498h[81]
$Pb(L\text{–}Br)_2$	2D	**2D – Topology 1**	(5,8)-c<br>$(3^4.4^4.5^2)(3^8.4^{10}.5^7.6^3)$	5-bromonicotinic acid	Pb^{2+}	FONXIS	2019	10.1016/j.jssc.2019.05.044[82]

$Pb(L–Cl)_2$	2D	**2D – Topology 1**	(5,8)-c $(3^{4}.4^{4}.5^{2})(3^{8}.4^{10}.5^{7}.6^{3})$	5-chloronicotinic acid	Pb^{2+}	ZOMQAW,<br>ZOMQAW1	2019,<br>2019	10.1016/j.molstruc.2019.05.031[83]<br>10.1016/j.jssc.2019.05.044[82]
$Mn(3\text{-}aba)_2$	2D	**2D – Topology 1**	(5,8)-c $(3^{4}.4^{4}.5^{2})(3^{8}.4^{10}.5^{7}.6^{3})$	3-amino benzoic acid	Mn^{2+}	UCUQEP	2006	10.1002/ejic.200500985[84]
$Cd(3\text{-}aba)_2$	2D	**2D – Topology 1**	(5,8)-c $(3^{4}.4^{4}.5^{2})(3^{8}.4^{10}.5^{7}.6^{3})$	3-amino benzoic acid	Cd^{2+}	MEDQUI	2006	10.5517/cc9t3v1[85]
$Co(3\text{-}aba)_2$	2D	**2D – Topology 1**	(5,8)-c $(3^{4}.4^{4}.5^{2})(3^{8}.4^{10}.5^{7}.6^{3})$	3-amino benzoic acid	Co^{2+}	MEGBOQ	2006	10.5517/ccb1g5z[86]
$Ni(3\text{-}aba)_2$	2D	**2D – Topology 1**	(5,8)-c $(3^{4}.4^{4}.5^{2})(3^{8}.4^{10}.5^{7}.6^{3})$	3-amino benzoic acid	Ni^{2+}	MEGBUW	2006	10.5517/ccb1g5z [86]
$Mn(C_8H_8NO_2)_2$	2D	**2D – Topology 1**	(5,8)-c $(3^{4}.4^{4}.5^{2})(3^{8}.4^{10}.5^{7}.6^{3})$	3-amino-p-toluic acid	Mn^{2+}	ULEZUI	2015	10.1134/S0022476615060165[87]
$Co(C_8H_8NO_2)_2$	2D	**2D – Topology 1**	(5,8)-c $(3^{4}.4^{4}.5^{2})(3^{8}.4^{10}.5^{7}.6^{3})$	3-amino-p-toluic acid	Co^{2+}	ULIBAU	2015	10.1134/S0022476615060165[87]
$Ni(C_8H_8NO_2)_2$	2D	**2D – Topology 1**	(5,8)-c $(3^{4}.4^{4}.5^{2})(3^{8}.4^{10}.5^{7}.6^{3})$	3-amino-p-toluic acid	Ni^{2+}	ULIBEY	2015	10.1134/S0022476615060165[87]
$Zn(C_8H_8NO_2)_2$	2D	**2D – Topology 1**	(5,8)-c $(3^{4}.4^{4}.5^{2})(3^{8}.4^{10}.5^{7}.6^{3})$	3-amino-p-toluic acid	Zn^{2+}	ULIBIC	2015	10.1134/S0022476615060165[87]
$Mn(3,4\text{-}daba)_2$	2D	**2D – Topology 1**	(5,8)-c $(3^{4}.4^{4}.5^{2})(3^{8}.4^{10}.5^{7}.6^{3})$	3,4-diamino benzoic acid	Mn^{2+}	HUWGEO	2020	10.1107/s2056989020006805[88]
$Cd(3\text{-}(3\text{-}(2\text{-}pyridin\text{-}4\text{-}yl\text{-}vinyl)phenyl)propenate)_2$	2D	**2D – Topology 1**	(5,8)-c $(3^{4}.4^{4}.5^{2})(3^{8}.4^{10}.5^{7}.6^{3})$	3-{3-(2-pyridin-4-yl-vinyl)-phenyl}-propenoic acid	Cd^{2+}	TIKWOA	2007	10.1021/cg060677j[34]
$Cd(Gly_3)_2$	2D	**2D – Topology 1**	(5,8)-c $(3^{4}.4^{4}.5^{2})(3^{8}.4^{10}.5^{7}.6^{3})$	μ_3-glycyl-glycyl-glycine	Cd^{2+}	MITROX	2008	10.1021/cg700724h[89]
$Mn(ima)_2$	2D	**2D – Topology 1**	(5,8)-c $(3^{4}.4^{4}.5^{2})(3^{8}.4^{10}.5^{7}.6^{3})$	2-(1H-imidazole-1-yl)acetic Acid	Mn^{2+}	JEXSAH,<br>JEXSAH01	2006,<br>2016	10.1071/CH06183[90]<br>CSD Communication
$Cd(ima)_2$	2D	**2D – Topology 1**	(5,8)-c $(3^{4}.4^{4}.5^{2})(3^{8}.4^{10}.5^{7}.6^{3})$	2-(1H-imidazole-1-yl)acetic Acid	Cd^{2+}	SEYVEY,<br>SEYVEY01	2007,<br>2016	10.1016/j.molstruc.2006.07.021[91]<br>CSD Communication
$Co(ima)_2$	2D	**2D – Topology 1**	(5,8)-c $(3^{4}.4^{4}.5^{2})(3^{8}.4^{10}.5^{7}.6^{3})$	2-(1H-imidazole-1-yl)acetic Acid	Co^{2+}	FUFYEL,<br>FUFYEL01,<br>FUFYEL02,<br>FUFYEL03	2009,<br>2009,<br>2010,<br>2012	10.1524/ncrs.2009.0212[92]<br>10.1016/j.jssc.2009.07.059[93]<br>10.1080/00958972.2010.481717[94]<br>10.1016/j.poly.2012.04.018[95]
$Fe(ima)_2$	2D	**2D – Topology 1**	(5,8)-c $(3^{4}.4^{4}.5^{2})(3^{8}.4^{10}.5^{7}.6^{3})$	2-(1H-imidazole-1-yl)acetic Acid	Fe^{2+}	FATRID	2012	10.1016/j.poly.2012.04.018[95]
$Ni(ima)_2$	2D	**2D – Topology 1**	(5,8)-c $(3^{4}.4^{4}.5^{2})(3^{8}.4^{10}.5^{7}.6^{3})$	2-(1H-imidazole-1-yl)acetic Acid	Ni^{2+}	JEXSEL	2006	10.1071/CH06183[90]
$Pb(ima)_2$	2D	**2D – Topology 1**	(5,8)-c $(3^{4}.4^{4}.5^{2})(3^{8}.4^{10}.5^{7}.6^{3})$	2-(1H-imidazole-1-yl)acetic Acid	Pb^{2+}	QAFQAR	2010	10.5517/cctypyc[96]
Mn((S)-imp)((R)-imp)*	2D	**2D – Topology 1**	(5,8)-c $(3^{4}.4^{4}.5^{2})(3^{8}.4^{10}.5^{7}.6^{3})$	(S/R)-2-(1H-imidazole-1-yl) propionic acid	Mn^{2+}	WUWVOA	2010	10.1021/cg1001368[97]
$Cd(tza)_2$	2D	**2D – Topology 1**	(5,8)-c $(3^{4}.4^{4}.5^{2})(3^{8}.4^{10}.5^{7}.6^{3})$	2-(1H-1,2,4-triazol-1-yl)acetic acid	Cd^{2+}	XOTTAC	2009	10.1016/j.ica.2008.06.013[98]
$Mn(tza)_2$	2D	**2D – Topology 1**	(5,8)-c $(3^{4}.4^{4}.5^{2})(3^{8}.4^{10}.5^{7}.6^{3})$	2-(1H-1,2,4-triazol-1-yl)acetic acid	Mn^{2+}	PEDVEB	2013	10.1016/j.molstruc.2012.07.016[99]
$Co(tza)_2$	2D	**2D – Topology 1**	(5,8)-c $(3^{4}.4^{4}.5^{2})(3^{8}.4^{10}.5^{7}.6^{3})$	2-(1H-1,2,4-triazol-1-yl)acetic acid	Co^{2+}	WUKFUF	2014	10.1515/ncrs-2014-0143[100]
$Cu(2\text{-}tza)_2$	2D	**2D – Topology 1**	(5,8)-c $(3^{4}.4^{4}.5^{2})(3^{8}.4^{10}.5^{7}.6^{3})$	2H-tetrazol-2-ylacetic acid	Cu^{2+}	PARPIK	2010	10.1016/j.inoche.2010.05.003[101]

Pb(4-pytza)$_2$	2D	**2D – Topology 1**	(5,8)-c $(3^4.4^4.5^2)(3^8.4^{10}.5^7.6^3)$	5-(4-pyridyl)tetrazole-2-acetic acid	Pb^{2+}	LUSYOP	2015	10.1039/C5RA17301A[102]
Pb(3-pytza)$_2$**†**	2D	**2D – Topology 1**	(5,8)-c $(3^4.4^4.5^2)(3^8.4^{10}.5^7.6^3)$	5-(3-pyridyl)tetrazole-2-acetic acid	Pb^{2+}	DOPKUQ	2014	10.1016/j.ica.2014.07.034[103]
Pb(pytac)$_2$	2D	**2D – Topology 1**	(5,8)-c $(3^4.4^4.5^2)(3^8.4^{10}.5^7.6^3)$	2-(4-pyridyl)thiazole-4-carboxylic acid	Pb^{2+}	NATVUB	2012	10.1007/s10870-011-0234-0[104]
Cd(DTBA)$_2$·H$_2$O	2D	**2D – Topology 1**	(5,8)-c $(3^4.4^4.5^2)(3^8.4^{10}.5^7.6^3)$	3,5-di(1H-1,2,4-triazol-1-yl)benzoic acid	Cd^{2+}	OGAPIY	2018	10.1039/C8CE01233D[105]
[Mn(3-cptpy)$_2$]$_n$	2D	**2D – Topology 1**	(5,8)-c $(3^4.4^4.5^2)(3^8.4^{10}.5^7.6^3)$	4′-(4-carboxyphenyl)-3,2′:6′,3″-terpyridine	Mn^{2+}	DIRMOI	2014	10.1039/C3DT52500G[106]
Cd(CO$_2$(CH$_2$)$_5$NH$_2$)$_2$	2D	**2D – Topology 1**	(5,8)-c $(3^4.4^4.5^2)(3^8.4^{10}.5^7.6^3)$	6-aminocaproic acid	Cd^{2+}	LAZNUW	2005	10.1002/chem.200500922[107]
Poly-[CdL$_2$]	2D	**2D – Topology 1**	(5,8)-c $(3^4.4^4.5^2)(3^8.4^{10}.5^7.6^3)$	N-(4-picolyl)-4-(40-carboxyphenoxy)1,8-naphthalimide	Cd^{2+}	DAQLOZ/ DAQLUF	2017	10.1039/C7CC03482B[108]
Ni(Haip)$_2$	2D	**2D – Topology 1**	(5,8)-c $(3^4.4^4.5^2)(3^8.4^{10}.5^7.6^3)$	5-aminoisophthalic acid	Ni^{2+}	ELOTAD	2021	10.1038/s41467-020-20489-2[109]
Mn(Haip)$_2$	2D	**2D – Topology 1**	(5,8)-c $(3^4.4^4.5^2)(3^8.4^{10}.5^7.6^3)$	5-aminoisophthalic acid	Mn^{2+}	ZOYJUU, ZOYJUU01, ZOYJUU02, ELOTEH/ELOSIK	2015, 2021, 2021, 2021	10.1021/ic502664e[110] 10.1038/s41467-020-20489-2[109] CSD Communication 10.1038/s41467-020-20489-2[109]
Co(Haip)$_2$	2D	**2D – Topology 1**	(5,8)-c $(3^4.4^4.5^2)(3^8.4^{10}.5^7.6^3)$	5-aminoisophthalic acid	Co^{2+}	WEMFEA, WEMFEA01, ELOSOQ	2006, 2021, 2021	10.1021/ic0602244[111] CSD Communication 10.1038/s41467-020-20489-2[109]
Co(HL)$_2$	2D	**2D – Topology 1**	(5,8)-c $(3^4.4^4.5^2)(3^8.4^{10}.5^7.6^3)$	4'-(pyridin-4-yl)-[1,1'-biphenyl]-3,5-dicarboxylic acid	Co^{2+}	CIDBEZ	2018	10.1039/C8CE00860D[112]
Mn(InMe-4-py)^2	2D	**2D – Topology 2**	(5,8)-c $(3^4.4^3.5^3)(3^8.4^9.5^8.6^3)$	2-(4-pyridylmethyl)-1,3-dioxoisoindoline-5-carboxylic acid	Mn^{2+}	YOQMOJ	2019	10.1016/j.jssc.2019.07.033[113]
Fe(pyoa)$_2$	2D	**2D – Topology 2**	(5,8)-c $(3^4.4^3.5^3)(3^8.4^9.5^8.6^3)$	2-(pyridin-3-yloxy)acetic acid	Fe^{2+}	VOJSUJ	2008	10.1021/ic701879y[114]
Cd(PyImPr)$_2$-**2D**	2D	**2D – Topology 1**	(5,8)-c $(3^4.4^4.5^2)(3^8.4^{10}.5^7.6^3)$	β-(3,4-pyridinedicarboximido)propionic acid	Cd^{2+}	2241486, 2241488	2023	**This paper**
Cd(PyImPr)$_2$-**hlz**	3D	**hlz**	(5,8)-c $(3^4.4^2.5^4)(3^8.4^8.5^6.6^5.7)$	β-(3,4-pyridinedicarboximido)propionic acid	Cd^{2+}	2241487, 2241489	2023	**This paper**

†Compounds with hemidirectional Pb^{2+}.

*Compounds involving racemic enantiomers.

**At the time of writing, the publication associated with NAFTIA mis-assigns atom types on the linker and so linker name was determined from the reaction procedure used.

References

1. P. Z. Moghadam, A. Li, S. B. Wiggin, A. Tao, A. G. P. Maloney, P. A. Wood, S. C. Ward and D. Fairen-Jimenez, *Chem. Mater.*, 2017, **29**, 2618-2625.
2. C. R. Groom, I. J. Bruno, M. P. Lightfoot and S. C. Ward, *Acta Crystallogr., Sect. B: Struct. Sci., Cryst. Eng. Mater.*, 2016, **72**, 171-179.
3. I. J. Bruno, J. C. Cole, P. R. Edgington, M. Kessler, C. F. Macrae, P. McCabe, J. Pearson and R. Taylor, *Acta Crystallogr., Sect. B: Struct. Sci., Cryst. Eng. Mater.*, 2002, **58**, 389-397.
4. N. Kumar, S.-Q. Wang, S. Mukherjee, A. A. Bezrukov, E. Patyk-Kaźmierczak, D. O'Nolan, A. Kumar, M.-H. Yu, Z. Chang, X.-H. Bu and M. J. Zaworotko, *Chem. Sci.*, 2020, **11**, 6889-6895.
5. V. A. Blatov, A. P. Shevchenko and D. M. Proserpio, *Cryst. Growth Des.*, 2014, **14**, 3576-3586.
6. S. S.-Y. Chui, S. M.-F. Lo, J. P. H. Charmant, A. G. Orpen and I. D. Williams, *Science*, 1999, **283**, 1148-1150.
7. A. Schoedel, M. Li, D. Li, M. O'Keeffe and O. M. Yaghi, *Chem. Rev.*, 2016, **116**, 12466-12535.
8. L. Shimoni-Livny, J. P. Glusker and C. W. Bock, *Inorg. Chem.*, 1998, **37**, 1853-1867.
9. R.-G. Xiong, S. R. Wilson and W. Lin, *J. Chem. Soc., Dalton Trans.*, 1998, 4089-4090.
10. H. D. Singh, S. Nandi, D. Chakraborty, K. Singh, C. P. Vinod and R. Vaidhyanathan, *Chem. - Asian J.*, 2022, **17**, e202101305.
11. Q. Wei, M. Nieuwenhuyzen, F. Meunier, C. Hardacre and S. L. James, *Dalton Trans.*, 2004, 1807-1811.
12. P. Pachfule, Y. Chen, J. Jiang and R. Banerjee, *Chem. - Eur. J.*, 2012, **18**, 688-694.
13. L. W.-H. HUA Xiao-Hui, XU Yi-Zhuang, WU Jin-Guang, *Acta Phys. -Chim. Sin.*, 2012, **28**, 1815-1822.
14. L. Zhu, N. Liu, L. Yu, X. Jiang and X. Li, *Inorg. Chim. Acta*, 2020, **510**, 119728.
15. T. Liu, D. Luo, D. Xu, H. Zeng and Z. Lin, *Dalton Trans.*, 2013, **42**, 368-371.
16. Y.-C. He, J. Guo, H.-M. Zhang, J. Yang, Y.-Y. Liu and J.-F. Ma, *CrystEngComm*, 2014, **16**, 4210-4214.
17. S. Nandi, P. De Luna, R. Maity, D. Chakraborty, T. Daff, T. Burns, T. K. Woo and R. Vaidhyanathan, *Mater. Horiz.*, 2019, **6**, 1883-1891.
18. P. Pachfule, B. Garai and R. Banerjee, *Inorg. Chem.*, 2016, **55**, 7200-7205.
19. D. Banerjee, H. Wang, A. M. Plonka, T. J. Emge, J. B. Parise and J. Li, *Chem. - Eur. J.*, 2016, **22**, 11816-11825.
20. R.-G. Lin, L. Li, R.-B. Lin, H. Arman and B. Chen, *CrystEngComm*, 2017, **19**, 6896-6901.
21. H. Wang, M. Warren, J. Jagiello, S. Jensen, S. K. Ghose, K. Tan, L. Yu, T. J. Emge, T. Thonhauser and J. Li, *J. Am. Chem. Soc.*, 2020, **142**, 20088-20097.
22. M. S. Zavakhina, D. G. Samsonenko, M. P. Yutkin, D. N. Dybtsev and V. P. Fedin, *Russ. J. Coord. Chem.*, 2013, **4**, 211.
23. S. Gao, R.-Q. Fan, L.-S. Qiang, P. Wang, S. Chen, X.-M. Wang and Y.-L. Yang, *CrystEngComm*, 2014, **16**, 1113-1125.
24. W.-W. Dong, J. Zhao and L. Xu, *Cryst. Growth Des.*, 2008, **8**, 2882-2886.
25. T. D. Keene, Y.-H. Deng, F.-G. Li, Y.-F. Ding, B. Wu, S.-X. Liu, C. Ambrus, O. Waldmann, S. Decurtins and X.-J. Yang, *Inorg. Chim. Acta*, 2009, **362**, 2265-2269.
26. D.-D. Feng, Y.-D. Zhao, X.-Q. Wang, D.-D. Fang, J. Tang, L.-M. Fan and J. Yang, *Dalton Trans.*, 2019, **48**, 10892-10900.
27. Q.-Q. Yan, B. Li and G.-P. Yong, *Polyhedron*, 2021, **194**, 114918.
28. X.-L. Nie, C.-Y. Hu, C.-G. Huang and X.-C. Shang-Guan, *Chin. J. Struct. Chem.*, 2015, **34**, 117-122.
29. Z.-H. Zhang and M. Du, *CrystEngComm*, 2008, **10**, 1350-1357.
30. R. Ding, P. Yan, G. Hou, P. Chen, Y. Wang and G. Li, *Synth. Met.*, 2012, **162**, 1894-1897.

31. S. Gao, R. Q. Fan, X. M. Wang, L. S. Qiang, L. G. Wei, P. Wang, H. J. Zhang, Y. L. Yang and Y. L. Wang, *J. Mater. Chem. A*, 2015, **3**, 6053-6063.
32. T. Yu, S. Wang, X. Li, X. Gao, C. Zhou, J. Cheng, B. Li, J. Li, J. Chang, H. Hou and Z. Liu, *CrystEngComm*, 2016, **18**, 1350-1362.
33. J.-W. Zhang, R.-J. Zhang, Y. Man, B.-Q. Liu and Y.-P. Dong, *J. Mol. Struct.*, 2019, **1185**, 276-280.
34. C.-D. Wu, P. Ayyappan, O. R. Evans and W. Lin, *Cryst. Growth Des.*, 2007, **7**, 1690-1694.
35. B. Wei, D. Xu, Z. Y. Yang, F. Sun, X. Q. Gu, C. Y. Sun, F. Y. Guo, Q. Y. Li and G. W. Yang, *J. Inorg. Organomet. Polym. Mater.*, 2017, **27**, 131-137.
36. L. Shen, Y. Bai, Y.-T. Min, T.-T. Jia, Q. Wu, J. Wang, F. Geng, H.-J. Cheng, D.-R. Zhu, J. Yang and G.-W. Yang, *J. Solid State Chem.*, 2016, **244**, 129-139.
37. H. Zhou, M. Li, D. Li, J. Zhang and X. Chen, *Sci. China: Chem.*, 2014, **57**, 365-370.
38. F. Guo, *Z. Kristallogr. - New Cryst. Struct.*, 2010, **636**, 857-860.
39. T. Liu, D. Luo, D. Xu, K. Wang and Z. Lin, *Inorg. Chem. Commun.*, 2014, **48**, 136-139.
40. G. Wang, *Synth. React. Inorg., Met.-Org., Nano-Met. Chem.*, 2013, **43**, 1311-1314.
41. H.-L. Zhou, R.-B. Lin, C.-T. He, Y.-B. Zhang, N. Feng, Q. Wang, F. Deng, J.-P. Zhang and X.-M. Chen, *Nat. Commun.*, 2013, **4**, 2534.
42. X. Chen, M.-X. Zhang, K.-L. Huang, F. Xiao and X.-P. Zhang, *Chin. J. Inorg. Chem.*, 2014, **33**, 1836-1842.
43. J.-Z. Gu, Z.-Q. Gao, D.-Y. Lv and W. Dou, *J. Chem. Crystallogr.*, 2012, **42**, 205-209.
44. W. Lin, M. E. Chapman, Z. Wang and G. T. Yee, *Inorg. Chem.*, 2000, **39**, 4169-4173.
45. X.-Y. Tian, H.-L. Zhou, X.-W. Zhang, C. Wang, Z.-H. Qiu, D.-D. Zhou and J.-P. Zhang, *Inorg. Chem.*, 2020, **59**, 6047-6052.
46. C.-P. Li, J. Chen, P.-W. Liu and M. Du, *CrystEngComm*, 2013, **15**, 9713-9721.
47. L.-N. Wang, L. Fu, J.-W. Zhu, Y. Xu, M. Zhang, Q. You, P. Wang and J. Qin, *Acta Chim. Slov.*, 2017, **64**, 6.
48. M. Chen, S.-S. Chen, T.-a. Okamura, Z. Su, M.-S. Chen, Y. Zhao, W.-Y. Sun and N. Ueyama, *Cryst. Growth Des.*, 2011, **11**, 1901-1912.
49. Y. J. Lee and S. W. Lee, *Polyhedron*, 2013, **53**, 103-112.
50. M. B. Cingi, A. g. Manfredotti, C. Guastini and A. Musatti, *Gazz. Chim. Ital.*, 1975, **105**, 117.
51. Z. R.-s. XU Xiao-yu, SONG Jiang-feng and X. Ji-qing, *Chem. Res. Chin. Univ.*, 2009, **25**, 279-281.
52. H. Sadeghzadeh and A. Morsali, *Ultrason. Sonochem.*, 2011, **18**, 80-84.
53. A. Rana, M. Bera, D. S. Chowdhuri, D. Hazari, R. J. Butcher and S. Dalai, *J. Inorg. Organomet. Polym. Mater.*, 2011, **21**, 747-753.
54. S. E.-d. H. Etaiw, D. M. Abd El-Aziz, H. Marie and E. Ali, *Solid State Sci.*, 2018, **79**, 15-22.
55. X.-S. Wu, Y.-R. Tang, J.-L. Liu, L. Wang and X.-M. Ren, *Dalton Trans.*, 2019, **48**, 13841-13849.
56. L. Yi and F. Guo, *Aust. J. Chem.*, 2020, **73**, 21-29.
57. X. Li, R. Cao, Y. Sun, W. Bi, X. Li and Y. Wang, *Eur. J. Inorg. Chem.*, 2005, **2005**, 321-329.
58. L.-X. Xie, X.-F. Zheng, H. Su and Q. Jin, *Acta Crystallogr., Sect. E: Struct. Rep. Online*, 2009, **65**, m1414.
59. D.-S. Liu, W.-T. Chen, Y.-P. Xu, P. Shen, S.-J. Hu and Y. Sui, *J. Solid State Chem.*, 2015, **226**, 186-191.
60. T. Yan, L. Du, L. Sun, X.-F. Zhang, T. Wang, J. Feng, J. Zhou and Q.-H. Zhao, *RSC Adv.*, 2017, **7**, 50150-50155.
61. B.-W. Hu, X.-Y. Zheng and C. Ding, *J. Solid State Chem.*, 2015, **232**, 62-66.
62. J. Chen, S.-H. Wang, Z.-F. Liu, M.-F. Wu, Y. Xiao, R. Li, F.-K. Zheng and G.-C. Guo, *Inorg. Chem. Commun.*, 2014, **46**, 207-211.
63. S.-J. Li, W.-D. Song, D.-L. Miao and D.-Y. Ma, *Chin. J. Struct. Chem.*, 2011, **30**, 1049-1053.
64. Z. Li, G. Zhang, T. Zhang, J. Zhang, L. Yang, Z. Zhou, S. Qi, K. Yu, F. Zhao, J. Yi, S. Xu and H. Gao, *Acta Chim. Sinica*, 2011, **69**, 1253-1258.

65. J. Li, G. Zhang, Y.-T. Li, X. Wang, J.-Q. Zhu and Y.-Q. Tian, *Open J. Inorg. Chem.*, 2012, **2**, 58-66.
66. A. A. García-Valdivia, E. Echenique-Errandonea, G. B. Ramírez-Rodríguez, J. M. Delgado-López, B. Fernández, S. Rojas, J. Cepeda and A. Rodríguez-Diéguez, *Inorganics*, 2021, **9**, 20.
67. Q. Y. Li, H. Tian, X. Y. Li, J. H. Zou, G. D. Mei, L. J. Qiu, B. Wei and G. W. Yang, *RSC Adv.*, 2015, **5**, 43741-43749.
68. G. W. Yang, Y. T. Zhang, Q. Wu, M. J. Cao, J. Wu, Q. Y. Yue and Q. Y. Li, *Inorg. Chim. Acta*, 2016, **450**, 364-371.
69. P. P. Sun, J. F. Dong, Y. Wang, Y. J. Liu, F. Sun, J. W. Yuan, G. W. Yang and Q. Li, *Inorg. Chem. Commun.*, 2016, **73**, 77-79.
70. L. Shen, M. J. Cao, F. F. Zhang, Q. Wu, L. Y. Zhao, Y. M. Lu, Q. Y. Li, G. W. Yang, B. Wei and J. H. Zou, *Transition Met. Chem.*, 2016, **41**, 125-131.
71. X.-J. Jiang, M. Du, Y. Sun, J.-H. Guo and J.-S. Li, *J. Solid State Chem.*, 2009, **182**, 3211-3214.
72. L. Tang, Y.-P. Wu, F. Fu, Q.-B. Wei and Q.-R. Lui, *Chin. J. Inorg. Chem.*, 2011, **27**, 2287- 2290.
73. C. Lin, W. Yu-Ling, L. Qing-Yan and Y. Yang, *Chin. J. Struct. Chem.*, 2016, **35**, 615-620.
74. B. Xu, Z. Guo, H. Yang, G. Li, T. Liu and R. Cao, *J. Mol. Struct.*, 2009, **922**, 140-143.
75. Y.-M. Song, R. Shi, X.-S. Wang, H. Zhao and R.-G. Xiong, *Appl. Organomet. Chem.*, 2005, **19**, 173-174.
76. Q.-X. Zhou, H.-F. Wang, X.-Q. Zhao, L. Yue and Y.-J. Wang, *Mater. Mech. Eng.*, 2004, **28**, 36-38.
77. W.-X. Yu, S.-F. Zhang and H.-B. Song, *J. Synth. Cryst.*, 2004, **33**, 384-387.
78. W.-W. Sun, C.-Y. Tian, Q. Yue and E.-Q. Gao, *J. Mol. Struct.*, 2009, **920**, 189-195.
79. K. C. Mondal, O. Sengupta, M. Nethaji and P. S. Mukherjee, *Dalton Trans.*, 2008, 767-775.
80. Y. Zhou, W. Xu, M. Wu, A. Lan, L. Zhang, R. Feng, F. Jiang and M. Hong, *Inorg. Chem. Commun.*, 2012, **15**, 140-145.
81. M. Du, X.-J. Zhao and Y. Wang, *Dalton Trans.*, 2004, 2065-2072.
82. F. Guo, C. Su, Y. Fan and W. Fu, *J. Solid State Chem.*, 2019, **277**, 83-92.
83. C. Miao, *J. Mol. Struct.*, 2019, **1193**, 286-293.
84. R. Wang, D. Yuan, F. Jiang, L. Han, S. Gao and M. Hong, *Eur. J. Inorg. Chem.*, 2006, **2006**, 1649-1656.
85. A.-Z. Tan, Y.-H. Wei, Z.-L. Chen, F.-P. Liang and R.-X. Hu, *Chin.J.Inorg.Chem.*, 2006, **22**, 394-398.
86. Y.-H. Wei, A.-Z. Tan, Z.-L. Chen and R.-X. Hu, *Struct. Chem.*, 2006, **25**, 343-347.
87. M. K. Khosa, P. T. Wood, S. M. Humphrey and W. T. A. Harrison, *J. Struct. Chem.*, 2015, **56**, 1130-1135.
88. M. K. Khosa, P. T. Wood, S. M. Humphrey and W. T. A. Harrison, *Acta Crystallogr., Sect. E: Crystallogr. Commun.*, 2020, **76**, 909-913.
89. H.-Y. Lee, J. W. Kampf, K. S. Park and E. N. G. Marsh, *Cryst. Growth Des.*, 2008, **8**, 296-303.
90. Y.-T. Wang, G.-M. Tang and D.-W. Qin, *Aust. J. Chem.*, 2006, **59**, 647-652.
91. Y.-T. Wang, G.-M. Tang, Y. Wu, X.-Y. Qin and D.-W. Qin, *J. Mol. Struct.*, 2007, **831**, 61-68.
92. Y.-T. Wang, R.-S. Xin and J.-G. Wang, *Z. Kristallogr. - New Cryst. Struct.*, 2009, **224**, 487-488.
93. B.-W. Hu, J.-P. Zhao, Q. Yang, T.-L. Hu, W.-P. Du and X.-H. Bu, *J. Solid State Chem.*, 2009, **182**, 2918-2923.
94. Y.-T. Wang, G.-M. Tang, Y.-C. Zhang, W.-Z. Wan, J.-C. Yu, T.-D. Li and Y.-Z. Cui, *J. Coord. Chem.*, 2010, **63**, 1504-1513.
95. H.-X. Chen, X.-P. Xu, Q.-F. Xu, Y. Zhang, H. Li, N.-J. Li, B. Wu and J.-M. Lu, *Polyhedron*, 2012, **41**, 77-85.
96. P. Liu, C.-C. Cao, Y.-P. Liang and Z.-S. Wu, *Chin. J. Inorg. Chem.*, 2010, **26**, 2057-2062.
97. G. Zhang, S.-Y. Yao, D.-W. Guo and Y.-Q. Tian, *Cryst. Growth Des.*, 2010, **10**, 2355-2359.
98. X.-Y. Zhou, Y.-Q. Huang and W.-Y. Sun, *Inorg. Chim. Acta*, 2009, **362**, 1399-1404.
99. X. Qian, P.-P. Sun, J.-G. Ding, B.-L. Li and H.-Y. Li, *J. Mol. Struct.*, 2013, **1031**, 175-179.

100. W.-F. Zhu and X. Zhu, *Z. Kristallogr. - New Cryst. Struct.*, 2014, **229**, 289-290.
101. S. V. Voitekhovich, T. V. Serebryanskaya, P. N. Gaponik, L. S. Ivashkevich, A. S. Lyakhov and O. A. Ivashkevich, *Inorg. Chem. Commun.*, 2010, **13**, 949-951.
102. G. W. Yang, F. F. Zhang, Q. Wu, M. J. Cao, Y. Bai, Q. Y. Li, B. Wei and J. H. Zou, *RSC Adv.*, 2015, **5**, 84439-84445.
103. J.-H. Zou, D.-L. Zhu, H. Tian, F. F. Li, F. F. Zhang, G.-W. Yang, Q.-Y. Li and Y. X. Miao, *Inorg. Chim. Acta*, 2014, **423**, 87-94.
104. R. C. Severance, A. M. Patel, M. D. Smith and H.-C. zur Loye, *J. Chem. Crystallogr.*, 2012, **42**, 258-262.
105. S.-Q. Deng, X.-J. Mo, Y. Feng, S.-L. Cai, W.-G. Zhang and S.-R. Zheng, *CrystEngComm*, 2018, **20**, 5531-5543.
106. P. Yang, M.-S. Wang, J.-J. Shen, M.-X. Li, Z.-X. Wang, M. Shao and X. He, *Dalton Trans.*, 2014, **43**, 1460-1470.
107. M. Dan and C. N. R. Rao, *Chem. - Eur. J.*, 2005, **11**, 7102-7109.
108. C. S. Hawes, A. D. Lynes, K. Byrne, W. Schmitt, G. Ryan, M. E. Möbius and T. Gunnlaugsson, *Chem. Commun.*, 2017, **53**, 5989-5992.
109. O. T. Qazvini, R. Babarao and S. G. Telfer, *Nat. Commun.*, 2021, **12**, 197.
110. C.-B. Tian, C. He, Y.-H. Han, Q. Wei, Q.-P. Li, P. Lin and S.-W. Du, *Inorg. Chem.*, 2015, **54**, 2560-2571.
111. E. Tang, Y.-M. Dai, J. Zhang, Z.-J. Li, Y.-G. Yao, J. Zhang and X.-D. Huang, *Inorg. Chem.*, 2006, **45**, 6276-6281.
112. L. Qin, Q. Hu, Y. Wu, J.-L. Cai and Y.-Y. Li, *CrystEngComm*, 2018, **20**, 4042-4048.
113. J.-Y. Wu, P.-T. Yuan, C.-C. Hsiao, H.-K. Chang, Y.-C. Liu, L.-J. Hsu and M.-H. Chiang, *J. Solid State Chem.*, 2019, **277**, 701-712.
114. Y.-Z. Zheng, W. Xue, M.-L. Tong, X.-M. Chen, F. Grandjean and G. J. Long, *Inorg. Chem.*, 2008, **47**, 4077-4087.
